# Comparative Analysis of *Isatis indigotica* Varieties Based on Integrated Metabolomic and Transcriptome Analyses

**DOI:** 10.3390/genes17091002

**Published:** 2026-08-25

**Authors:** Dong Liu, Jinjin Meng, Tao Du, Xiangsong Meng, Pan Wang

**Affiliations:** 1 School of Chinese Materia Medica and Health, Bozhou University, Bozhou 236800, China; liudong804214@163.com (D.L.);; 2 School of Pharmacy, Gansu University of Chinese Medicine, Chengguan District, Lanzhou 730000, China; 3 School of Pharmacy, Gansu Health Vocational College, Lanzhou New Area, Lanzhou 730207, China

**Keywords:** *Isatis indigotica* Fort, multi-omics, cultivar difference, photosynthesis, flavonoid metabolism, medicinal ingredients

## Abstract

Objectives: *Isatis indigotica* is a core medicinal crop for producing Radix *Isatidis* and Folium *Isatidis*, although cultivar genotype severely affects medicinal quality. This study aimed to systematically dissect the phenotypic, physiological, metabolomic, and transcriptomic differences between two cultivars, Songming No. 1 (SL, new elite line) and Dinglan No.1 (DL, conventional cultivar), and uncover the molecular mechanisms underlying SL’s superior agronomic and medicinal traits. Methods: Agronomic phenotypes, photosynthetic pigments, antioxidant enzyme activities, and leaf indirubin content were measured across seven growth stages (A–G). Untargeted LC-MS/MS metabolomics analyses were conducted at stages A, B, and E, and full-stage transcriptome sequencing was performed followed by qRT-PCR validation. Multi-omics correlation was visualized via nine-quadrant analysis. Results: SL exhibited a significantly larger leaf size, higher leaf biomass, and persistently elevated indirubin concentration at all stages. Its chlorophyll, carotenoid, Calvin-cycle enzyme, and AsA-GSH antioxidant enzyme activities were mostly higher than those in DL. A total of 1217 metabolites and massive differentially expressed genes (DEGs) were identified; flavonoids, especially peonidin-3-glucoside, stably accumulated in SL and mapped to anthocyanin biosynthesis. Transcriptional variations between cultivars concentrated on chloroplast pathways, with two candidate genes, *LOC18994026* and *LOC104719239*, potentially boosting SL photosynthesis. Seventeen differential metabolites correlated with 170 DEGs, while DL showed stronger cold tolerance linked to tryptophan-synthesis gene overexpression, and SL was inferred to be more sensitive to high light. Conclusions: In conclusion, SL possesses comprehensive advantages in yield, photosynthetic performance, and medicinal ingredient accumulation, serving as an excellent germplasm for large-scale cultivation. This multi-omics study clarifies genotypic divergence mechanisms and provides theoretical support for high-quality *I. indigotica* breeding.

## 1. Introduction

*Isatis indigotica* Fort., a widely cultivated medicinal Brassicaceae species in China, is valued for its roots (Banlangen), leaves (Daqingye), and processed products (Qingdai) in traditional medicine [1]. Its leaf extracts are also applied as natural plant-derived dyes [2,3,4]. Multiple bioactive constituents, dominated by indigo and indirubin, have been characterized in *I. indigotica* leaves [5]. As the principal bioactive ingredient, indirubin exerts anti-inflammatory, antiviral, and antitumor bioactivities [6,7,8]. Preparations derived from *I. indigotica* roots and leaves have shown promising efficacy for the prevention and clinical treatment of COVID-19 [9,10].

Phenotypic and metabolic polymorphisms are commonly observed among *I. indigotica* germplasms, and genotypic differences between cultivars substantially shape agronomic performance and secondary-metabolite profiles [11,12]. Such inherent varietal heterogeneity is one major source of inconsistent quality for medicinal raw materials, even when plants are cultivated under uniform environmental conditions. Although previous studies have illustrated how growing conditions alter indirubin and flavonoid contents in *I. indigotica*, most available reports emphasize environmental effects rather than genotype-dependent metabolite accumulation. Consequently, the genetic and metabolic regulatory networks that underpin cultivar-specific biosynthesis and accumulation of key pharmacologically active compounds (e.g., indirubin) remain largely uncharacterized. To fill this important knowledge gap, it is critical to dissect varietal divergence from both a physiological and molecular perspective, which will underpin future breeding efforts and support the standardized production of this high-value medicinal crop.

After long-term artificial cultivation, the agronomic traits and medicinal constituents of *I. indigotica* vary substantially across growing regions, which causes inconsistent quality across medicinal raw materials and unstable clinical therapeutic effects. Therefore, breeding high-yielding, high-quality, and stable cultivars is critical to guaranteeing uniform medicinal quality for production. For many crop species, comparative studies between cultivars have demonstrated that varietal selection should match diverse production objectives, and multi-omics integration has become a powerful tool to dissect intraspecific differences, characterize quality-related regulatory networks, and support rational germplasm utilization [13,14,15,16]. Nevertheless, such multi-omics-driven varietal evaluation remains limited for *I. indigotica*, despite its high medicinal–economic value.

Existing publications concerning *I. indigotica* mainly focus on four directions: the biosynthetic regulation of active pharmaceutical ingredients; gene-family identification; gene-function validation and miRNA-mediated regulatory mechanisms; and the pharmacological assessment of plant extracts [17,18,19]. Previous works have uncovered key genes participating in indirubin biosynthesis, floral transition, stamen development, and stress responses of *I. indigotica*. However, most of these studies were performed using single-genotype materials; few investigations have systematically deciphered phenotypic, metabolic, and transcriptomic divergence between improved *I. indigotica* cultivars. Specifically, the molecular basis underlying the cultivar-specific accumulation of core bioactive compounds (e.g., indirubin and flavonoids) is still poorly resolved, which hinders targeted breeding and variety popularization of this medicinal herb.

“Songming No. 1” (SL) is an elite *I. indigotica* cultivar that our research group systematically developed via multi-generation field screening from 2015 to 2023; it represents the first Chinese medicinal herb cultivar granted new-plant-variety protection rights in China. “Dinglan No. 1” (DL) is a mainstream commercial cultivar bred by the Dingxi Academy of Agricultural Sciences (Gansu, China) and is widely used as a reference control in regional *I. indigotica* germplasm evaluations. Field observations indicate that SL exhibits vigorous vegetative growth, enhanced drought tolerance, and improved resistance to root-rot disease (disease-resistance grade T). However, these field surveys only focused on agronomic and stress-resistant phenotypes; whether SL also possesses advantages in the biosynthesis and accumulation of core pharmacodynamic metabolites relative to DL has not yet been clarified.

Rationale for selecting SL and DL for this comparative study: The two cultivars represent typical genetic resources for *I. indigotica* production: a newly released elite breeding line versus a conventional, widely planted commercial check cultivar. Obvious phenotypic distinctions can be observed under identical field cultivation conditions, making them ideal materials to dissect genotypic-driven varietal differences while minimizing confounding environmental noise. Scientific hypothesis: Genotypic divergence between SL and DL drives multi-layer physiological, metabolic, and transcriptomic variations. The superior agronomic performance of SL is accompanied by reprogrammed secondary-metabolite networks, especially for flavonoids and indirubin. These phenotypic–metabolic disparities are governed by key differentially expressed genes predominantly involved in photosynthesis and secondary-metabolite biosynthetic pathways.

For medicinal crop breeding, yield and stress-resistance performance are essential indicators; equally important is the evaluation of metabolite profiles that determine medicinal quality. Accordingly, the present study adopted combined phenotypic, metabolomic, and transcriptomic strategies to systematically compare SL and DL regarding photosynthetic pigment contents, pharmacodynamic-ingredient accumulation, secondary-metabolite profiles, and transcriptomic landscapes. This work aims to unravel the superior agronomic and medicinal traits of SL and their underlying molecular regulatory mechanisms, and provide solid theoretical support for the large-scale cultivation, industrial processing, and popularization of this promising new cultivar.

## 2. Materials and Methods

### 2.1. Plant Materials and Cultivation Conditions

“Songming No. 1” (SL) is an *I. indigotica* cultivar systematically selected and preserved by our research group. “Dinglan No. 1” (DL) was bred and provided by the Dingxi Academy of Agricultural Sciences, Gansu, China. Seed materials were taxonomically authenticated by Professor Du Tao, and voucher specimens of SL and DL were deposited in the herbarium of Gansu University of Chinese Medicine (Voucher No.: GZYSL-songming1hao-20240801; GZYSL-songming1hao-20240802).

Field experiments were carried out at Hezheng Medicinal Botanical Garden, Gansu University of Chinese Medicine (Hezheng County, Gansu, China; 35°15′48″ N, 103°24′21″ E; altitude 2430 m). The site has a temperate continental monsoon climate, with an annual average precipitation of 660 mm, frost-free period of 110 days, mean annual temperature of 5.1 °C, annual maximum temperature of 29.0 °C, and annual minimum temperature of −20.4 °C. The experimental soil is black loessal soil, and the experimental field was fallow in the previous cropping season. SL and DL seeds were sown on 23 May 2024 in rows with 30 cm row spacing and sowing depth of 3–5 cm. At the six-true-leaf seedling stage, seedlings were thinned to 10 cm plant-to-plant spacing. Identical field management practices (watering, weeding, fertilization, and pest control) were applied uniformly for both cultivars throughout the entire growing season.

### 2.2. Sample Collection

Seven sampling stages (A–G) were set for this study. The sampling timeline was based on the Chinese 24-Solar-Terms calendar; however, developmental stages were primarily defined by unified morphological indicators (leaf number, plant height, and rosette status) rather than calendar date alone to ensure that SL and DL were harvested at a comparable phenological status and to avoid developmental noise caused by possible differences in growth rate between cultivars. Days after emergence and morphological criteria corresponding to each stage (A–G) are listed in Table 1.

For each cultivar at each developmental stage, three biological replicates were collected. For each biological replicate, 10 individual healthy plants were randomly selected from the field. Above-ground tissues were harvested, roots were discarded, and the four outermost fully expanded leaves per plant were excised at the leaf base. Leaf materials were rinsed to remove surface soil and blotted dry with absorbent filter paper, and their main veins were removed. Leaf tissues from 10 plants within one replicate were mixed to form one composite sample. Fresh subsamples were immediately frozen in liquid nitrogen and stored at −80 °C for metabolomic, transcriptomic, and physiological measurements. The remaining leaf materials were cleaned, air-dried indoors in shade, ground, and passed through a 60-mesh sieve to obtain dry powder samples for indirubin quantification. Sample codes for each time-point are presented in Table 1.

### 2.3. Measurement of Morphological Characteristics

Ten SL plants and ten DL plants were randomly selected to observe leaf surface characteristics and measure plant height, plant width, leaf length, leaf width, and leaf number. After harvesting, the fresh leaf weight of each plant was determined, and the dry weight was measured after air-drying in shade.

### 2.4. Determination of Photosynthetic Pigment Content

Ten SL plants and ten DL plants were randomly selected. The four outermost leaves were excised at the base of each plant and rinsed with distilled water, and the main veins were removed. Subsequently, leaves were cut into 0.1–0.2 mm strips to determine chlorophyll a, chlorophyll b, and carotenoid contents. Pigment extraction was performed using the acetone method [20], and absorbance was measured using a UV spectrophotometer (Shimadzu, Kyoto, Japan). Pigment concentrations were calculated based on the Beer–Lambert law [21]. Chlorophyll a and b were collectively referred to as total chlorophyll.

### 2.5. Determination of Photosynthetic Physiological Indices

Leaf samples from the two cultivars collected during the seven stages were used to determine photosynthetic physiological indices. Assay kits from Shanghai Enzyme-Linked Biotechnology Co., Ltd., (Shanghai, China) were used to measure the activities of ascorbate peroxidase (APX), glutathione reductase (GR), glutathione peroxidase (GSH-Px), superoxide dismutase (SOD), ribulose-1,5-bisphosphate carboxylase/oxygenase (Rubisco), sedoheptulose-1,7-bisphosphatase (SBPase), fructose-1,6-bisphosphate aldolase (FBA), and transketolase (TK), together with the contents of reduced glutathione (GSH) and oxidized glutathione (GSSG).

### 2.6. Determination of Indirubin Content

Indirubin content in leaf dry powder was quantified via high-performance liquid chromatography (HPLC) [22,23].

Standard solution preparation: An indirubin reference substance was dissolved in a methanol–chloroform mixed solvent (*v*/*v* = 4:1) to prepare a stock standard solution. Serial dilutions were performed to establish the standard curve.

Sample extraction: Leaf dry powder was extracted with chloroform under reflux. After cooling and filtration, the solvent was removed under reduced pressure. Residues were re-dissolved with methanol–chloroform (4:1), transferred to volumetric flasks, and filtered prior to HPLC injection.

Chromatographic conditions: Agilent 5TC-C18 (Agilent, Santa Clara, CA, USA) column (4.6 mm × 250 mm); mobile phase: acetonitrile (A) and ultrapure water (B); gradient elution: 0–12 min 20% A; 12–24 min 20–30% A; 24–36 min 30–40% A; 36–50 min 40–55% A; flow rate = 1.0 mL·min^−1^; column temperature = 25 °C; injection volume = 10 μL; and detection wavelength = 289 nm.

The standard regression equation was y = 37.32x + 170.76 (*R*^2^ = 0.9998), with a linear range of 0.0625–1 μg·mL^−1^.

### 2.7. Metabolomics Detection and Analysis

According to the dynamic pattern of indirubin accumulation across the seven developmental stages, stages A, B, and E were selected for non-targeted metabolomic profiling, representing the rosette vigorous-expansion (A), rapid vegetative-growth (B), and peak secondary-metabolite accumulation (E) stages, respectively. Frozen leaf samples (−80 °C storage, three biological replicates per cultivar per stage) were subjected to LC-MS/MS-based metabolomics analysis performed by Shanghai Applied Protein Technology Biotechnology Co., Ltd. (APT-BIO, Shanghai, China). Sample processing, LC-MS/MS acquisition, and quality control procedures followed the company’s standard workflow and previously reported protocols [24].

Multivariate statistical analysis, differential metabolite screening, and KEGG pathway enrichment were performed. Differential metabolites were screened with the following criteria: |log_2_FC| ≥ 1, VIP ≥ 1.

### 2.8. Transcriptome Sequencing and RT-qPCR Validation

#### 2.8.1. Transcriptome Sequencing

Total RNA was isolated from leaf samples of SL and DL cultivars across the seven developmental stages. RNA concentration and purity were measured using a NanoDrop 2000 spectrophotometer (Thermo Scientific, Wilmington, DE, USA). RNA degradation and preliminary integrity were assessed via 1% agarose-gel electrophoresis, and the RNA quality number (RQN) was determined using an Agilent 5300 system(Agilent Technologies Inc., Santa Clara, CA, USA) to further evaluate RNA integrity. Messenger RNA was enriched using oligo-dT magnetic beads and fragmented with fragmentation buffer. First-strand cDNA was reverse-transcribed from fragmented mRNA templates, followed by second-strand cDNA synthesis. Adapter sequences were ligated to double-stranded cDNA fragments. Adapter-ligated products were purified and size-selected, and the size-fractionated fragments were amplified using PCR. Purified qualified cDNA libraries were finally obtained and subjected to high-throughput sequencing.

Raw sequencing reads were pre-processed to remove adapters and low-quality reads to generate clean reads for subsequent analyses. Since no publicly available reference genome of *I. indigotica* exists, clean reads were assembled de novo using Trinity (v2.15.2) software to produce a set of unigenes that served as reference transcripts. Clean reads were mapped back to the assembled unigene sequences. Gene expression abundance was calculated as fragments per kilobase of transcript per million mapped reads (FPKM). Differential-expression analysis was performed using the DESeq2 R package (v1.42.0). Transcripts meeting the criteria |log_2_FC| ≥ 2 and *p* < 0.05 were defined as differentially expressed genes (DEGs). Functional annotation of unigenes was carried out against public databases including GO and KEGG.

#### 2.8.2. qRT-PCR Validation

Four differentially expressed transcripts were selected for RT-qPCR validation, with Actin used as the reference gene. Quantitative PCR primers were designed accordingly, and all primer sequences are listed in Table 2. Purified total RNA was reverse-transcribed into cDNA using the Evo M-MuLV First Strand cDNA Synthesis Kit, and fluorescence quantitative analysis was carried out using a rapid quantitative PCR mix kit. The genomic DNA removal reaction was performed in a 10 μL system and incubated at 42 °C for 2 min. First-strand cDNA synthesis was conducted in a 20 μL reaction system. Real-time PCR was performed in a total volume of 20 μL, containing 10 μL of 2 × SYBR Green Pro Taq HS Premix, 0.4 μL Primer F (10 μM), 0.4 μL Primer R (10 μM), and 0.4 μL ROX Reference Dye (20 μM). The amplification protocol consisted of an initial pre-denaturation at 95 °C for 30 s, followed by 40 cycles of 95 °C for 5 s and 60 °C for 30 s. Relative gene expression levels were calculated using the 2^−ΔΔCt^ method.

### 2.9. Metabolome and Transcriptome Correlation Analysis

Multi-omics correlation analysis was carried out to link DEGs and differential metabolites. Pearson correlation coefficients were calculated between transcript abundance of DEGs and metabolite intensities using R software(v4.3.2). Pairs with |*r*| > 0.6 and *p* < 0.05 were regarded as significantly correlated. Nine-quadrant analysis was implemented to visualize the correlation patterns between transcripts and metabolites.

## 3. Results

### 3.1. Morphological Traits

Morphological characteristics provided the most intuitive visual distinction between the two varieties. Leaf morphological observations indicated that SL leaves were obovate, wrinkled, and slightly waxy, with slightly notched or entire moderately undulated margins. In contrast, DL leaves were lanceolate and flat with moderate waxiness, slight marginal notches, and mild undulation at the leaf base. Other leaf traits were comparable between varieties.

**Figure 1:**
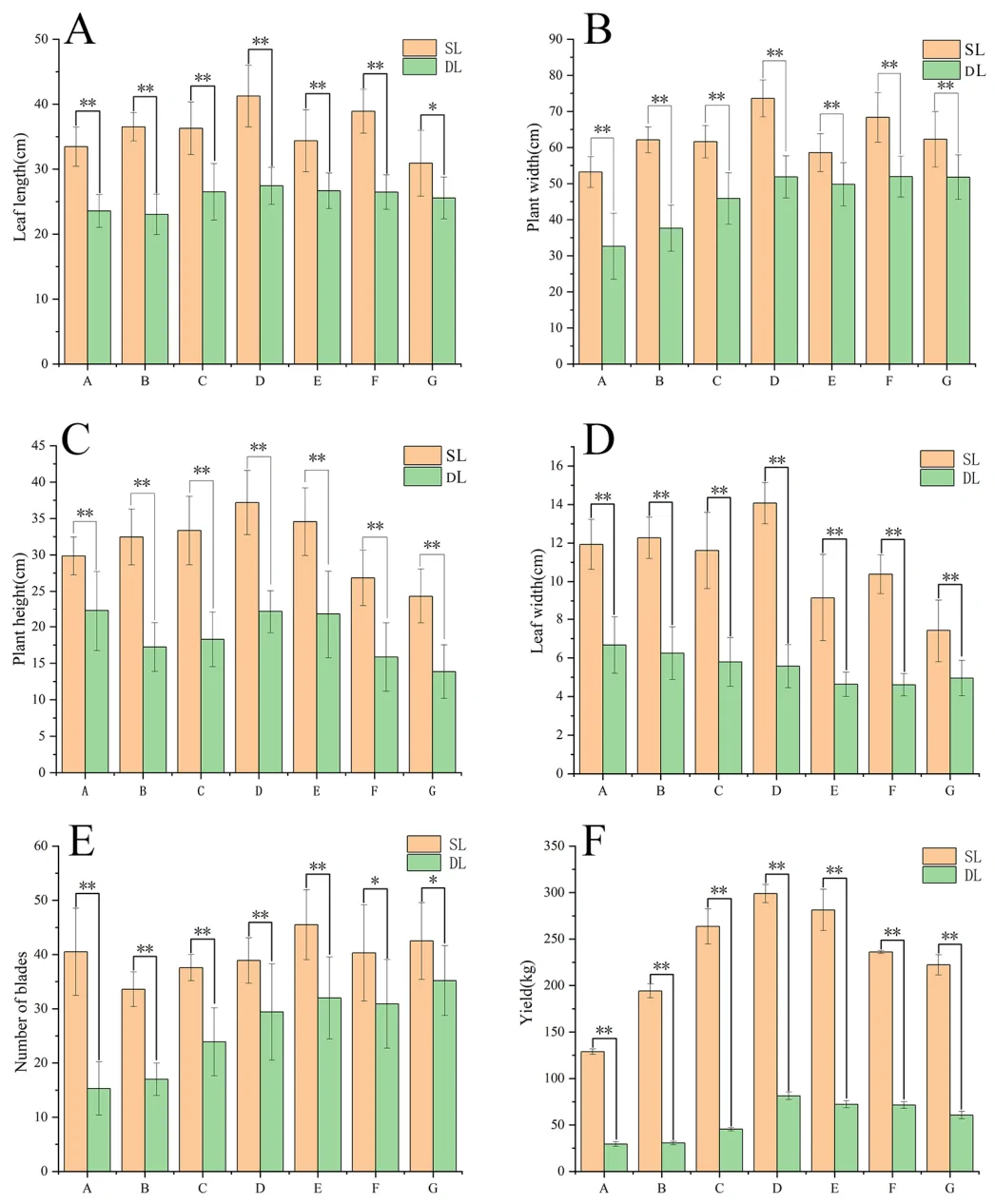

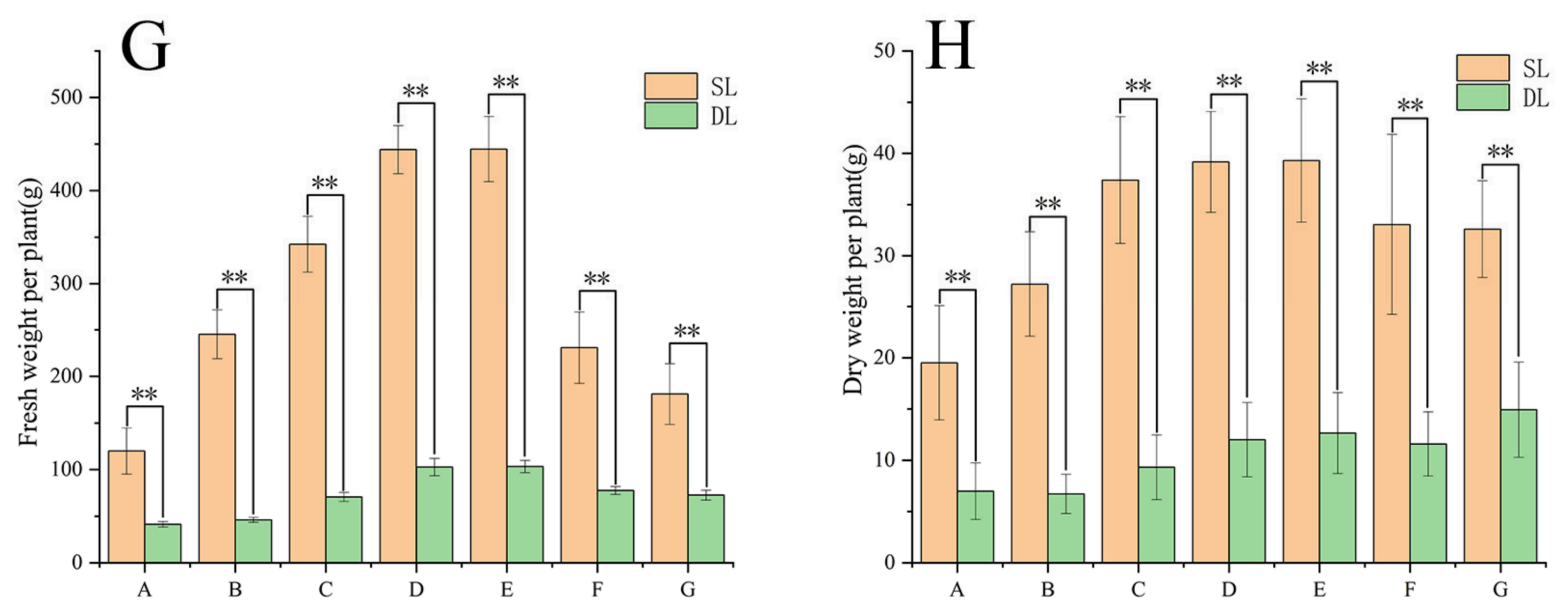
Measurements of SL and DL agronomic indicators during seven stages: leaf length (**A**), plant width (**B**), plant height (**C**), leaf width (**D**), number of leaves (**E**), yield (**F**), fresh weight per plant (**G**), and dry weight per plant (**H**). The *x*-axis indicates the time of sampling, and the *y*-axis indicates the result of the measured indexes. * denotes significant difference (*p* < 0.05); ** denotes difference is very significant (*p* < 0.01).

Agronomic trait measurements conducted at the seven developmental stages (Figure 1) demonstrated that SL had a significantly higher plant height, plant width, leaf length, leaf width, and leaf number relative to DL, suggesting SL showed better leaf morphology. Meanwhile, SL maintained a significantly higher leaf fresh weight per plant, leaf dry weight per plant, and yield per mu (1 mu = 0.0667 ha) throughout all seven sampling stages.

### 3.2. Photosynthetic Pigment Content and Physiological Indicators

#### 3.2.1. Photosynthetic Pigment Content

**Figure 2:**
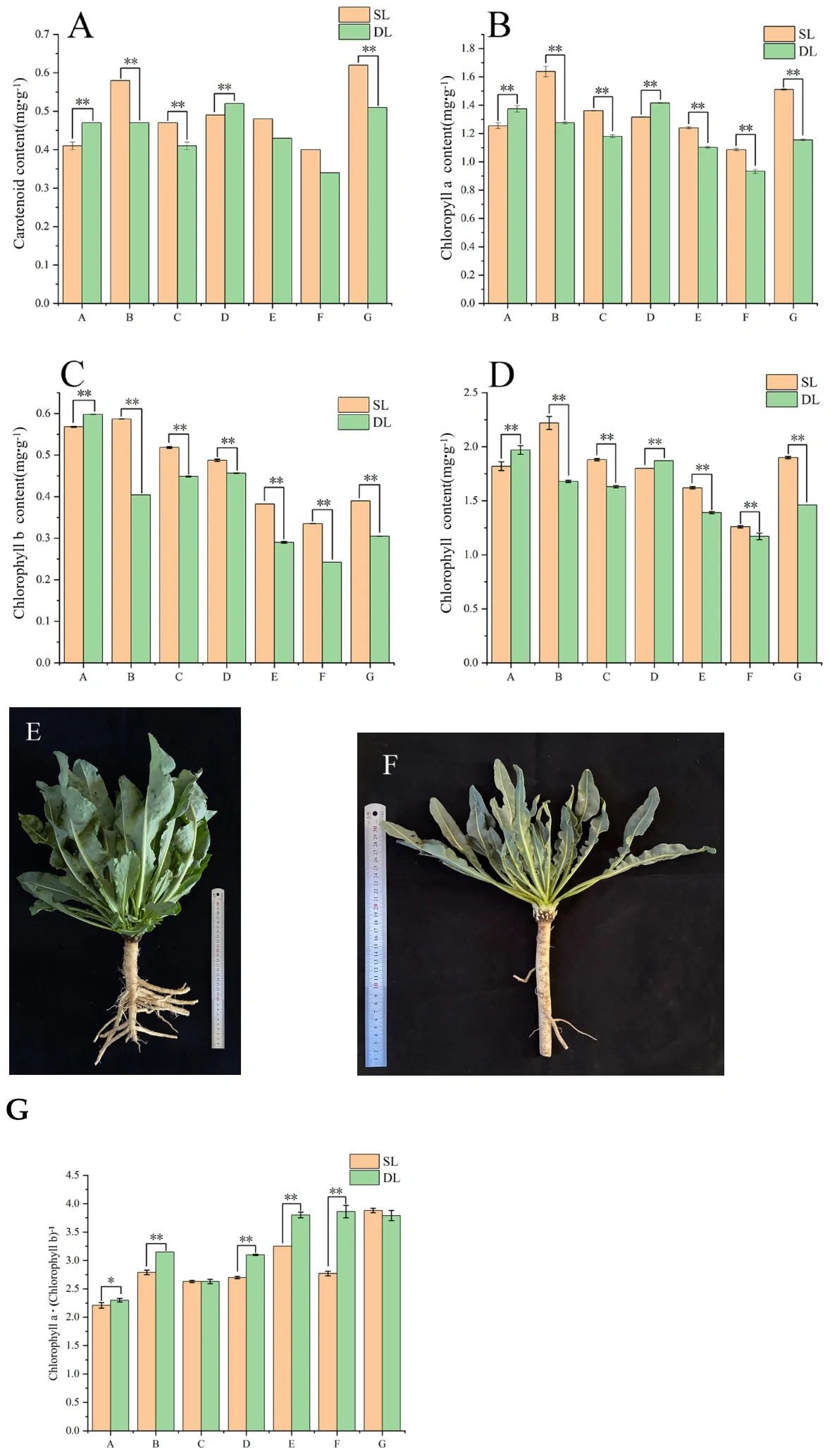
Dynamic changes in leaf photosynthetic pigments of SL and DL across seven developmental stages. (**A**) Carotenoid content; (**B**) chlorophyll a content; (**C**) chlorophyll b content; (**D**) total chlorophyll content; (**E**) original photographs of SL plant morphology at stage E; (**F**) original photographs of DL plant morphology at stage E; (**G**) chlorophyll a/b ratio. The *x*-axis represents the seven successive sampling stages, and the *y*-axis represents the quantitative value of each physiological index. Data are presented as mean ± standard error. * denotes significant difference at *p* < 0.05; ** denotes highly significant difference at *p* < 0.01.

The dynamic accumulation patterns of leaf photosynthetic pigments (chlorophyll a, chlorophyll b, total chlorophyll, and carotenoids) in SL and DL cultivars were quantified across the seven developmental stages, with significant temporal and cultivar-specific variations observed (Figure 2). The chlorophyll a and chlorophyll b contents of SL leaves exhibited a consistent dynamic trend: increasing from stage A to B, declining continuously from B to F, and recovering slightly at stage G, with significant differences detected among developmental stages. By contrast, DL chlorophyll a content decreased from A to C, increased from C to D, declined from D to F, and finally rebounded at stage G. DL chlorophyll b presented a similar fluctuation pattern throughout development.

The total chlorophyll content results further verified the differential accumulation characteristics between the two cultivars. For SL, total chlorophyll peaked at stage B (2.22 mg/g) and reached the minimum level at stage F (1.26 mg/g). For DL, the maximum chlorophyll content occurred at stage A (1.97 mg/g), while the minimum value was observed at stage F (1.17 mg/g). Comparative analysis indicated that DL possessed significantly higher chlorophyll accumulation than SL only at stage A, whereas SL exhibited substantially higher total chlorophyll levels during all subsequent developmental stages (B–G).

Leaf carotenoid accumulation also displayed distinct temporal variations between cultivars. SL carotenoid content increased notably from A to B, decreased gradually from B to F with a mild decline during stages C–E, and recovered at stage G. In DL leaves, carotenoid content decreased from A to C, increased from C to D, decreased again from D to F, and finally increased at stage G.

#### 3.2.2. Photosynthetic Physiological Indicators

**Figure 3:**
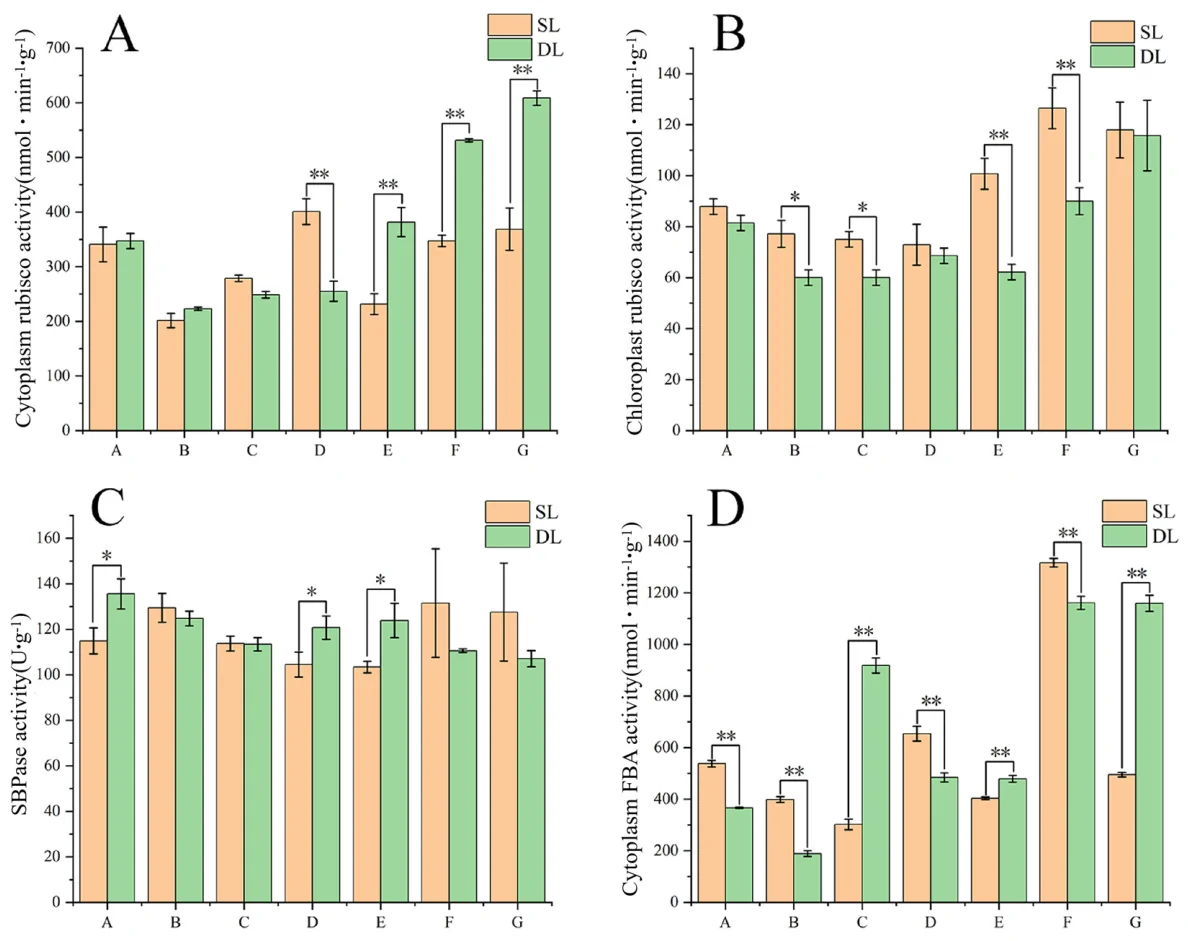

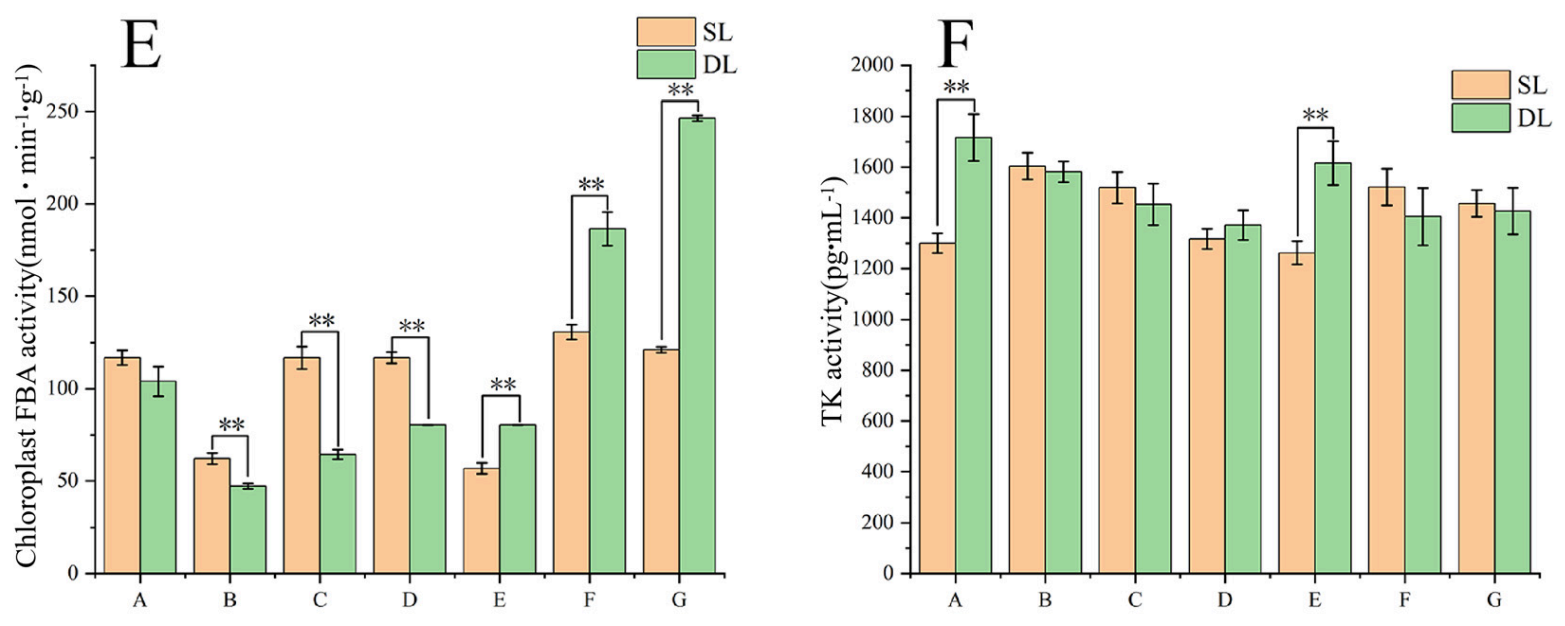
Measurement of photosynthetic physiological indicators across seven stages of SL and DL: cytoplasm Rubisco activity (**A**), chloroplast Rubisco activity (**B**), SBPase activity (**C**), cytoplasm FBA activity (**D**), chloroplast FBA activity (**E**), TK activity (**F**). * denotes significant difference (*p* < 0.05); ** denotes difference is very significant (*p* < 0.01).

Determinations of Rubisco activity in the cytoplasm (Figure 3A) and chloroplasts (Figure 3B) from SL and DL leaves showed that cytoplasmic Rubisco activity in SL leaves was superior to DL only at stages C and D but markedly lower at stages E, F, and G. In contrast, chloroplast Rubisco activity was continuously higher in SL relative to DL. SBPase activity (Figure 3C) was significantly greater in SL leaves than in DL at stages B, F, and G. Cytoplasmic FBA activity (Figure 3D) was significantly higher in SL than in DL at stages D and F only, whereas chloroplastic FBA activity (Figure 3E) remained consistently higher in SL throughout all sampling stages. TK activity (Figure 3F) in both cultivars presented a general pattern of decreasing, increasing, and subsequently decreasing.

**Figure 4:**
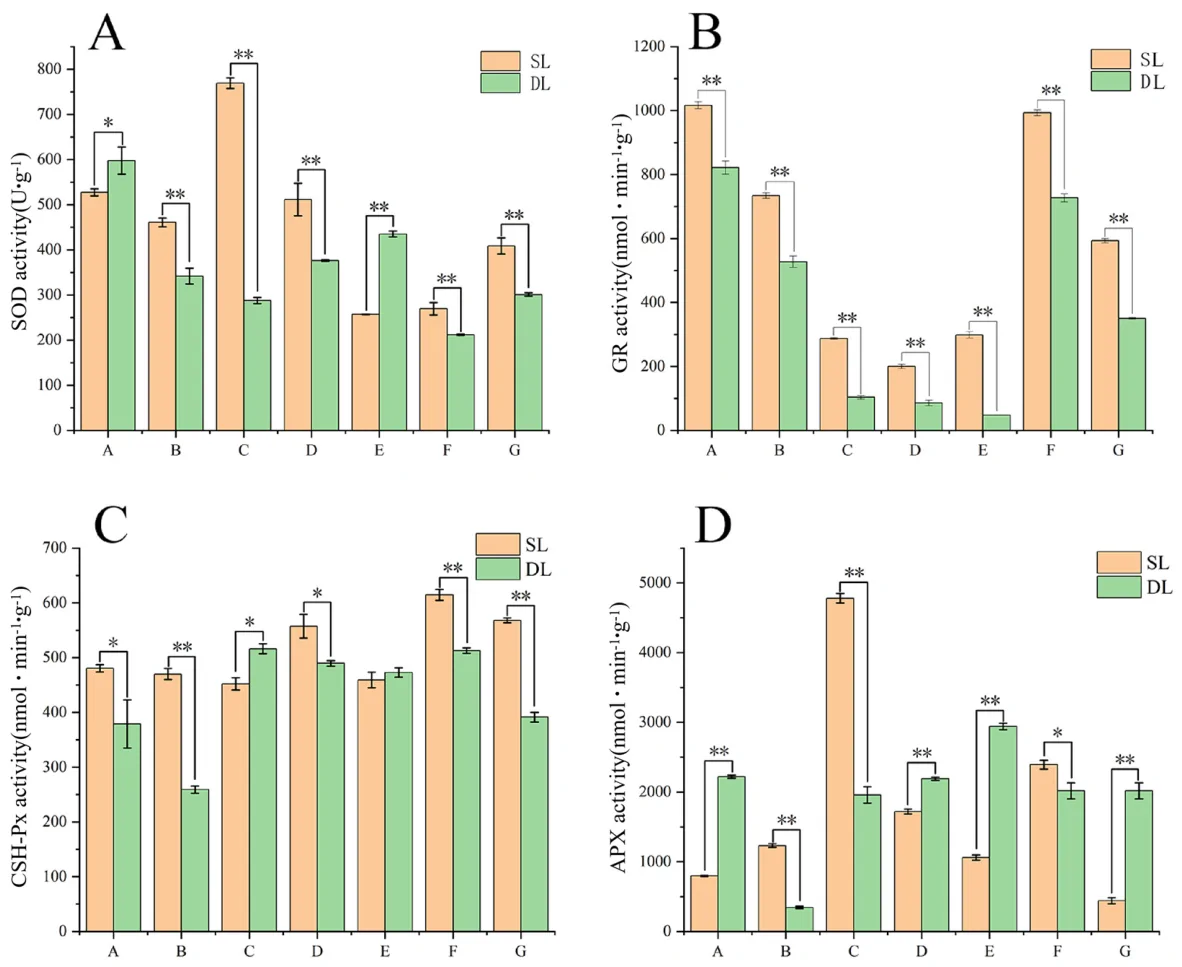

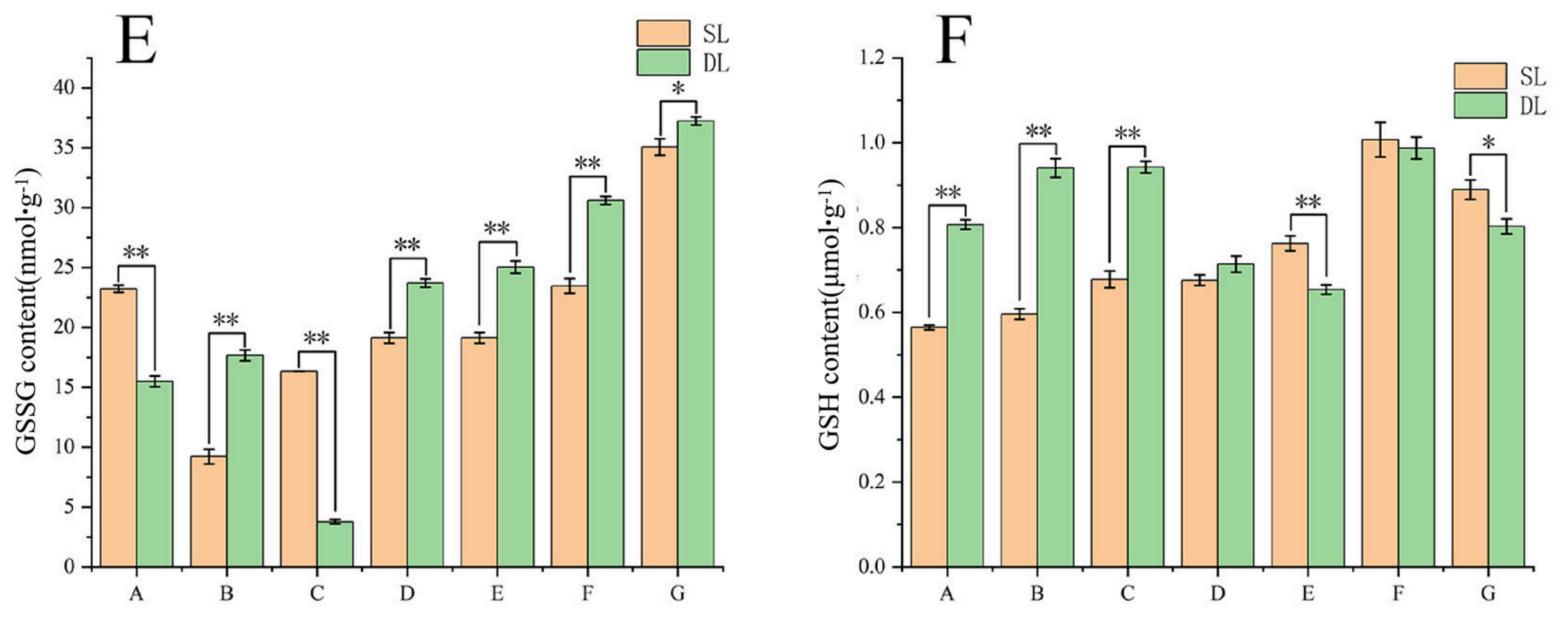
Measurement of photosynthetic physiological indicators across seven stages of SL and DL: SOD activity (**A**), GR activity (**B**), GSH-Px activity (**C**), APX activity (**D**), GSSG content (**E**), GSH content (**F**). * denotes significant difference (*p* < 0.05); ** denotes difference is very significant (*p* < 0.01).

The ascorbic acid–glutathione (AsA-GSH) cycle is vital for plant tolerance against various environmental stresses. SOD activity (Figure 4A) was significantly higher in SL leaves than in DL at stages B, C, D, F, and G, and APX activity (Figure 4D) was markedly greater in SL at stages B, C, and F. GR activity (Figure 4B) in both cultivars showed an overall decreasing–increasing–decreasing trend and remained higher in SL than DL across all seven stages. GSH-Px activity (Figure 4C) was elevated in SL relative to DL at stages A, B, D, F, and G. SL leaves possessed a higher GSSG content (Figure 4E) at stages A and C, whereas a greater GSH content (Figure 4F) was observed in SL at stages E, F, and G.

### 3.3. Indirubin Content

**Figure 5:**
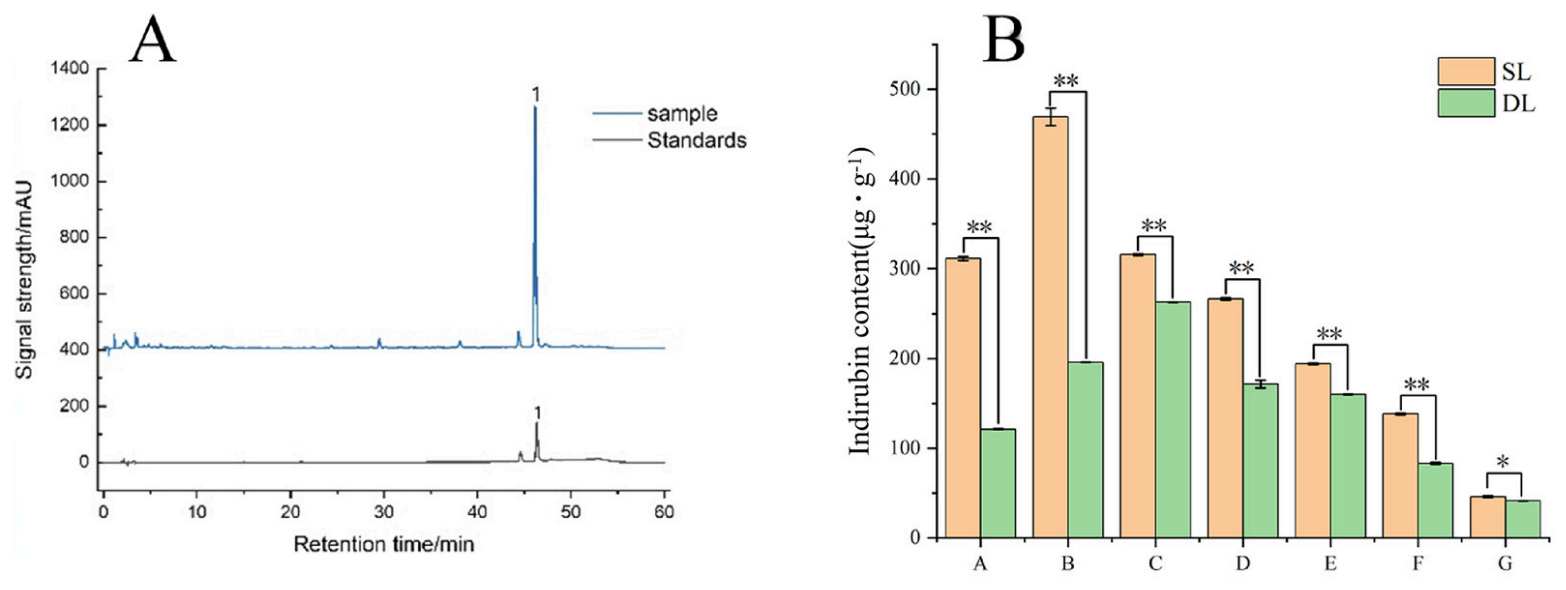
SL and DL indirubin content. HPLC chromatogram of sample extracts (**A**), Indirubin content at different developmental stages of two cultivars (**B**). * denotes significant difference (*p* < 0.05); ** denotes difference is very significant (*p* < 0.01).

Indirubin, an indole alkaloid with anti-inflammatory, antiviral, and antitumor activities, is the principal bioactive constituent in *Isatis* leaves. HPLC analysis was performed to quantify the indirubin contents in SL and DL leaves at the seven developmental stages (Figure 5B). Indirubin concentrations in both cultivars first increased and subsequently decreased. SL attained the highest level at stage B (469.35 ppm), while the maximum value for DL appeared at stage C (263.07 ppm). Indirubin content was significantly greater in SL relative to DL throughout all seven stages, and the largest disparities were detected at stages A and B.

### 3.4. Metabolomic Analysis

#### 3.4.1. Metabolome Annotation and Classification Based on Non-Targeted Metabolomics

**Figure 6:**
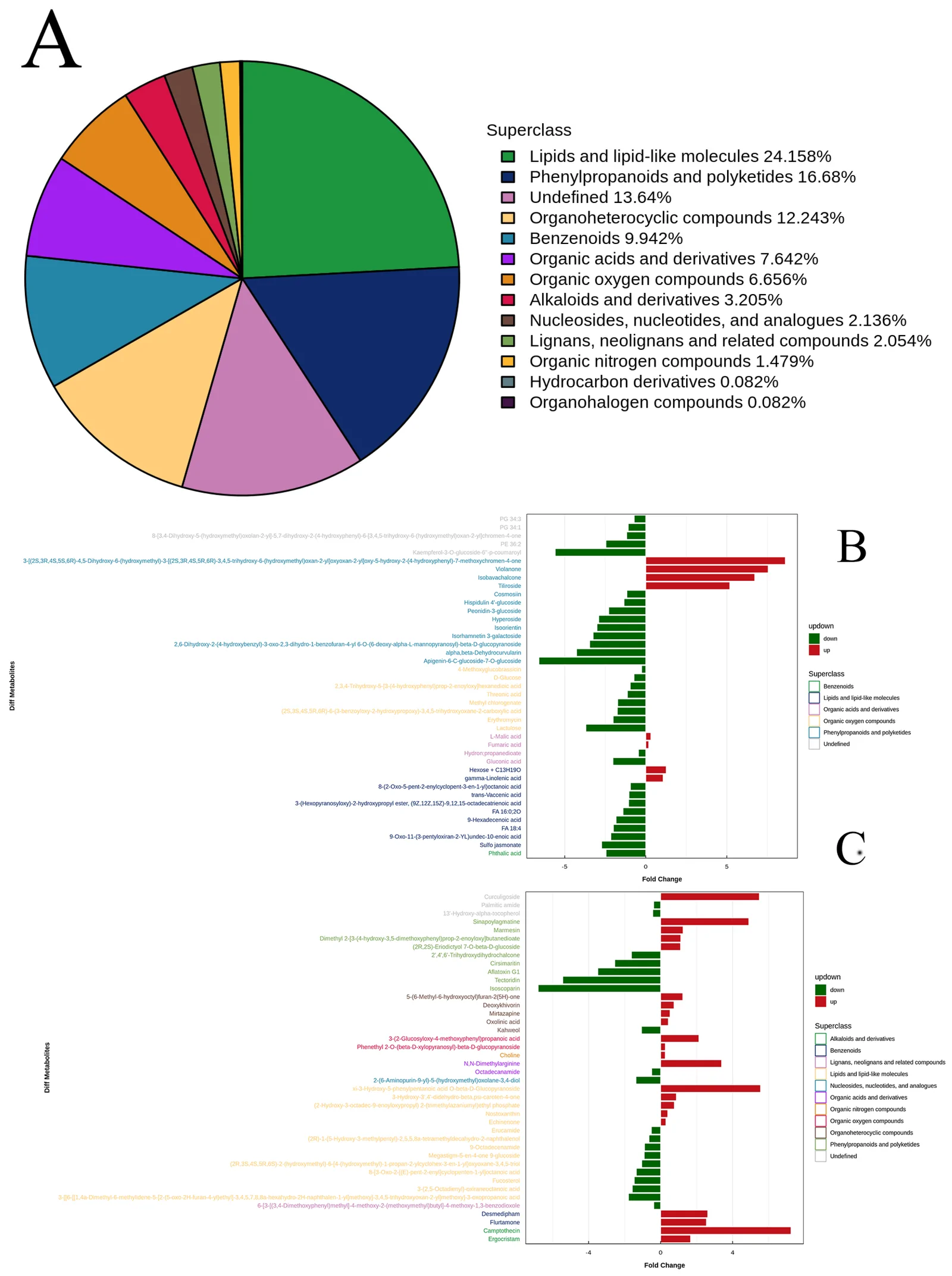

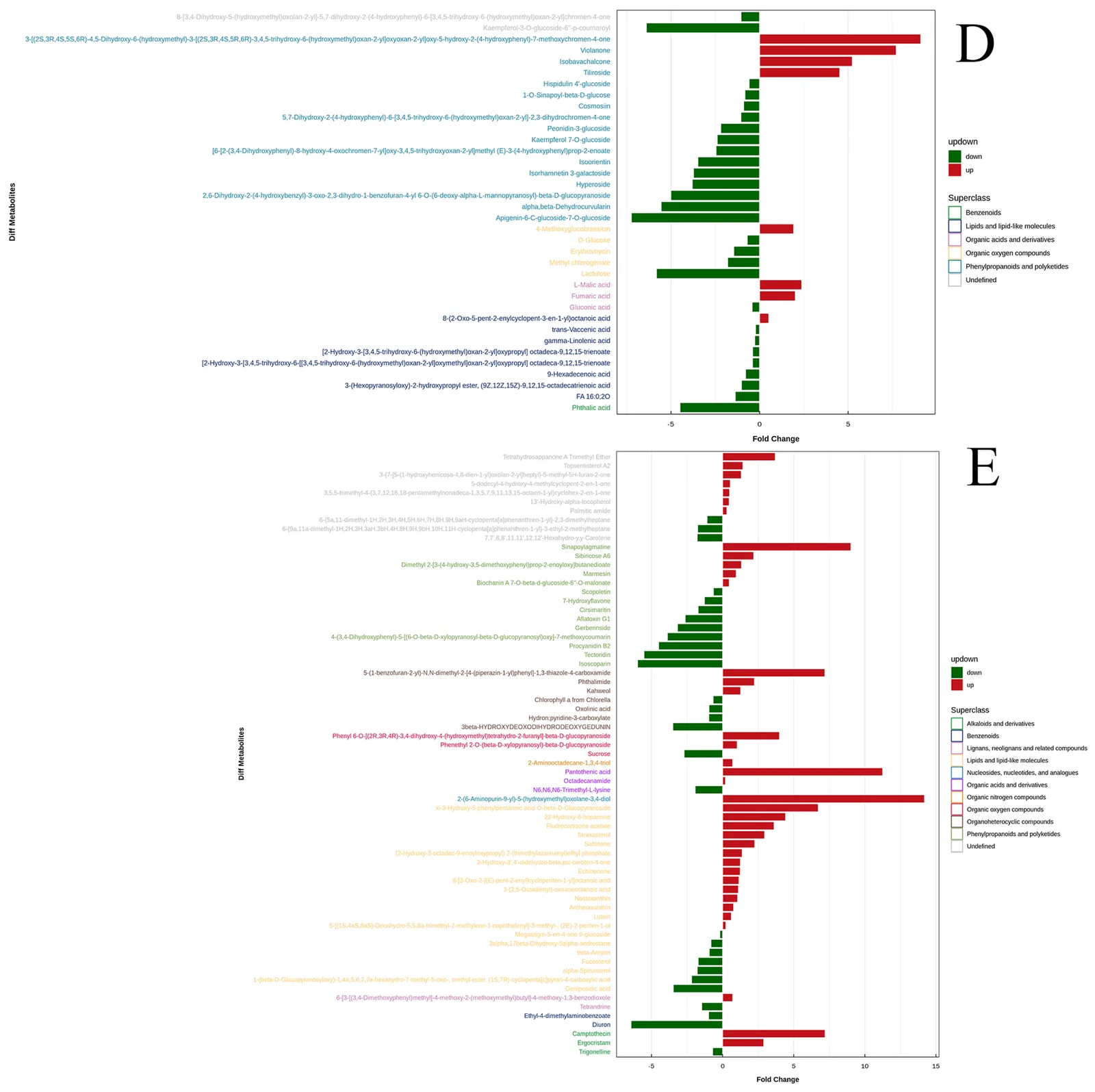

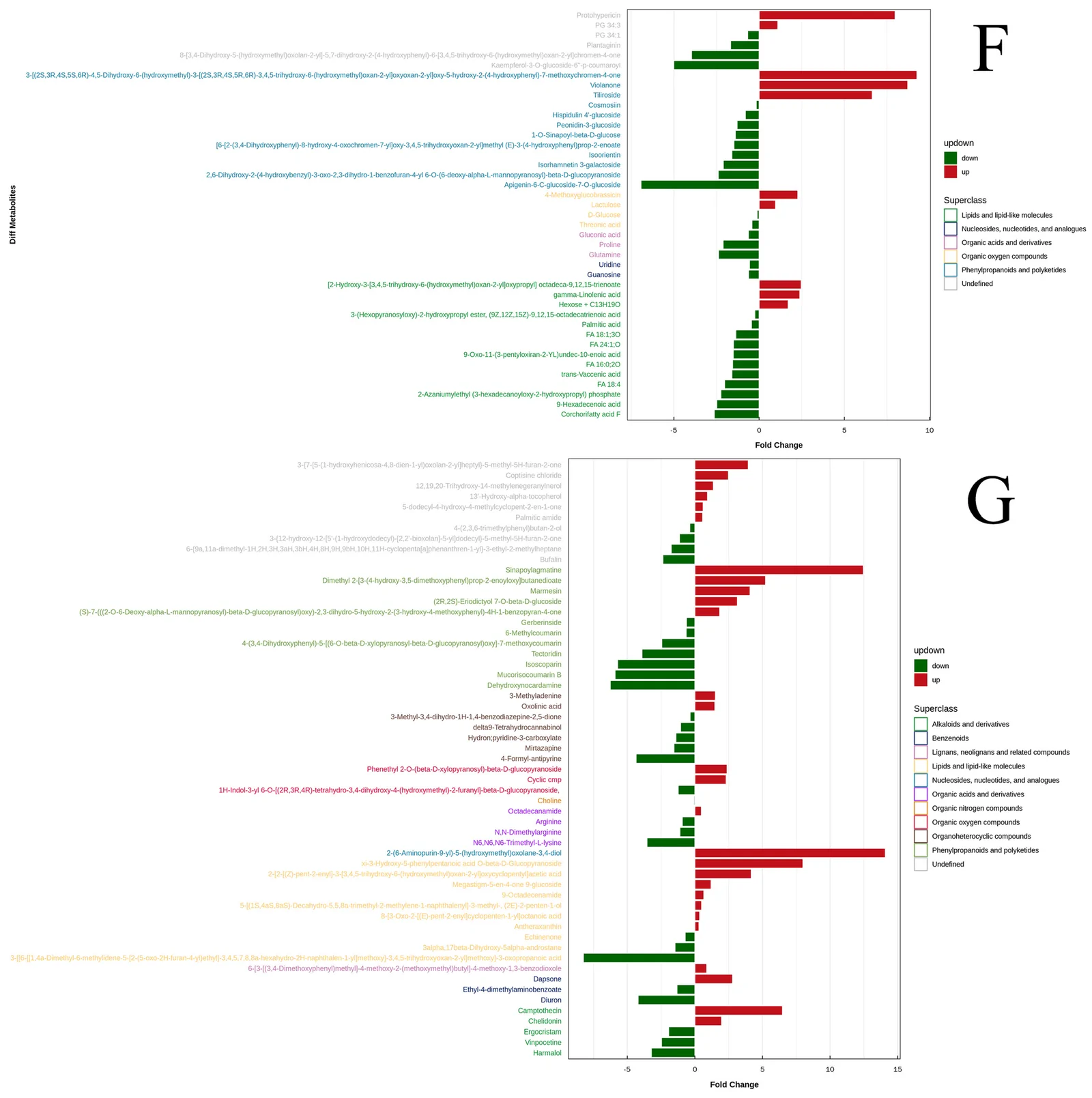
Plot of metabolomics data for stages (**A**,**B**). (**A**,**E**) are a classification of metabolite identification results, (**B**) is the result of the NEG mode in stage (**A**), (**C**) is the result of the POS mode in stage (**A**), (**D**) is the result of the NEG mode in stage (**B**), (**E**) is the result of the POS mode in stage (**B**), (**F**) is the result of the NEG mode in stage E, and (**G**) is the result of the POS mode in stage E.

In total, 1217 metabolites were successfully annotated and categorized into 13 major classes (Figure 6A), dominated by lipids and lipid-like molecules (24.16%), phenylpropanoids and polyketides (16.68%), and unclassified metabolites (13.64%). Other annotated categories included organoheterocyclic compounds, benzenoids, organic acids and derivatives, and other minor metabolite classes.

Ion mode classification was used to show that 515 metabolites were detected in negative ionization mode, including indirubin (NEG3865), the core bioactive ingredient of *I. indigotica* leaves. A total of 702 metabolites were identified in positive ionization mode, covering indigo (POS3010), another key functional component of *Isatis* leaves. All metabolomic analyses were performed based on samples collected at three representative developmental stages (A, B, and E), corresponding to the rosette vigorous-expansion stage, rapid vegetative-growth stage, and secondary-metabolite peak accumulation stage, respectively.

#### 3.4.2. Differential Metabolite Identification and Profiling

Differential metabolite analysis between SL and DL was conducted at three key developmental stages (A, B, and E) under both positive (POS) and negative (NEG) ionization modes to reveal cultivar-specific metabolic differences.

In POS ionization mode, cultivar-based metabolic differences exhibited obvious temporal specificity. At stage A, a total of 41 DMs were identified between SL and DL (Figure 6B), among which 8 metabolites were significantly accumulated and 33 were depleted in SL. Notably, all differentially accumulated organic oxygen compounds (eight metabolites) were uniformly downregulated in SL. At stage B, 36 DMs were screened, including 8 upregulated and 28 downregulated metabolites in SL, with the sole detected benzenoid metabolite showing complete suppression. At stage E, 41 DMs were characterized, consisting of 10 elevated and 31 reduced metabolites in SL. Specifically, all differential organic acid derivatives (three metabolites) and nucleotide analogs (two metabolites) displayed decreased abundance in SL relative to DL.

In NEG ionization mode, distinct metabolic variations were also observed across developmental stages. Forty-two DMs were detected at stage A (Figure 6A), with 22 metabolites increased and 20 decreased in SL. The enriched metabolites in SL mainly included organic oxygen compounds, organic nitrogen compounds, benzenoids, and alkaloid derivatives, whereas nucleotide and lignan-related metabolites were significantly depleted. At stage B, 67 DMs were identified, of which 38 were upregulated and 29 were downregulated in SL. Organic nitrogen compounds and nucleotide metabolites were induced in SL, while benzenoid metabolites were exclusively suppressed. At stage E, 57 DMs were obtained, with 29 metabolites upregulated and 28 downregulated; notably, nucleotide and lignan derivatives were consistently enriched in SL at this stage.

Cross-stage dynamic analysis further screened constitutively differential metabolites throughout the key developmental periods. In POS mode, 21 metabolites exhibited stable differential accumulation across stages A, B, and E, primarily dominated by flavonoids (10 members), followed by fatty acyls and organic oxygen compounds. Among these persistent DMs, three metabolites were continuously upregulated and 15 were downregulated in SL during development. In NEG mode, 18 constitutively altered metabolites were identified, covering diverse classes including fatty acyls, cinnamic acid derivatives, alkaloids, and terpenoids. Six metabolites were persistently elevated and two were continuously reduced in SL across all three stages.

Integrated analysis of POS and NEG metabolomic datasets identified 26 core DMs that stably differentiated the metabolic profiles of SL and DL, including 9 consistently upregulated and 17 downregulated metabolites in SL. Flavonoids represented the most predominant differential chemical class, and the core annotated metabolites included 9-hexadecenoic acid, cosmosiin, D-glucose, gluconic acid, isoorientin, kaempferol-3-O-glucoside-6′′-pcoumaroyl, peonidin-3-glucoside, tiliroside, transvaccenic acid, violanone, isoscoparin, echinenone, marmesin, sinapoylagmatine, tectoridin, and camptothecin. Metabolic pathway annotation revealed that peonidin-3-glucoside and violanone were specifically assigned to pathways C12141 and C10195, respectively.

### 3.5. Transcriptome Analysis

#### 3.5.1. Transcriptome Sequencing and De Novo Assembly

Transcriptome sequencing was performed on SL and DL leaf samples across the seven developmental stages (three biological replicates per group, 42 samples in total) to explore the molecular basis of cultivar divergence. A total of 295.2 G clean data was generated, with each sample yielding over 5 Gb high-quality reads. The Q30 ratio exceeded 96% and the GC content was above 46% for all samples, confirming qualified sequencing data for subsequent assembly and analysis. Due to the absence of a publicly available *I. indigotica* reference genome, clean reads were assembled de novo using Trinity software. Assembly statistics showed that the transcript N50 and N90 were 1780 bp and 391 bp, while the unigene N50 and N90 were 1049 bp and 267 bp, respectively. Most unigene sequences ranged from 0 to 600 bp, with only a small proportion longer than 3000 bp, reflecting a typical unigene length distribution of a de novo assembled transcriptome.

#### 3.5.2. Functional Annotation of Unigenes

All assembled unigenes were functionally annotated against five public databases (NR, Pfam, Swiss-Prot, KEGG, and GO). In total, 253,221 unigenes were successfully annotated, among which 30,332 were annotated in the GO database, and 4965 unigenes obtained unified annotation across all five databases. GO classification analysis showed that “cellular process” (20,148 unigenes) dominated the biological process category, “binding” (18,206 unigenes) was the most abundant term in molecular function, and “cell” (22,617 unigenes) was predominant in the cellular component category. NR database alignment indicated that the annotated unigenes were mostly matched to Mus musculus (24.34%), followed by Eutrema salsugineum (16.02%) and Brassica rapa (11.03%), reflecting the evolutionary homology of isatis genes with cruciferous species.

#### 3.5.3. Identification and Functional Analysis of DEGs

**Figure 7:**
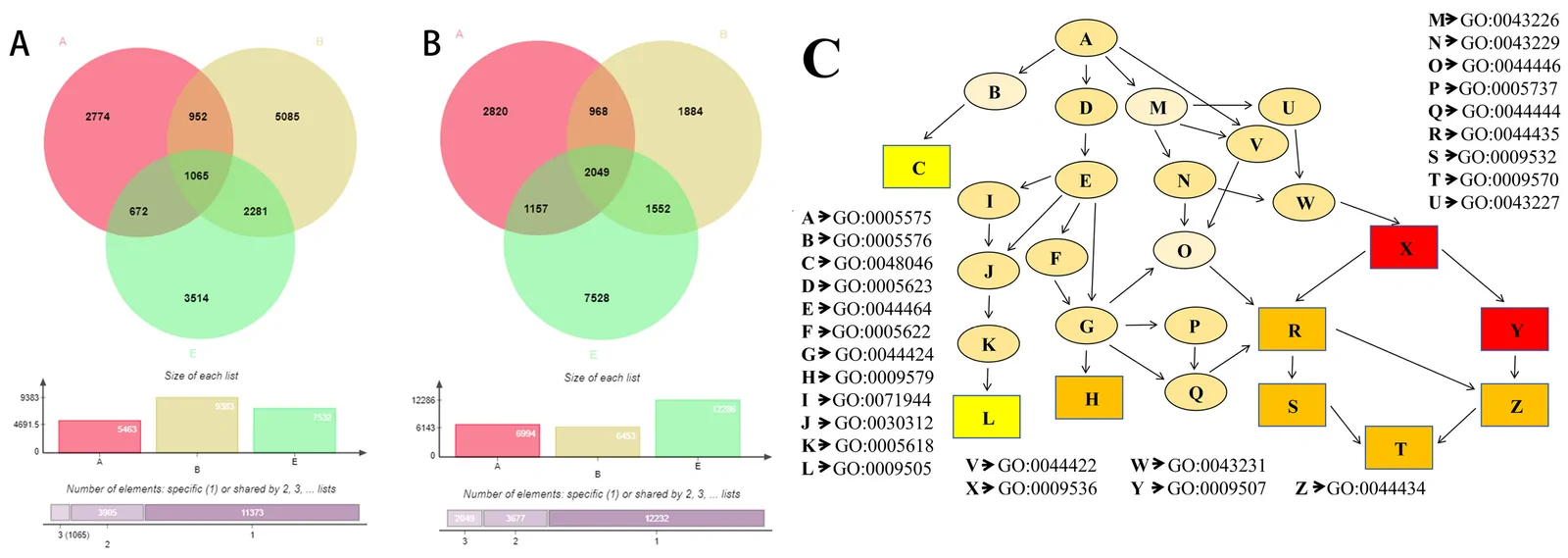

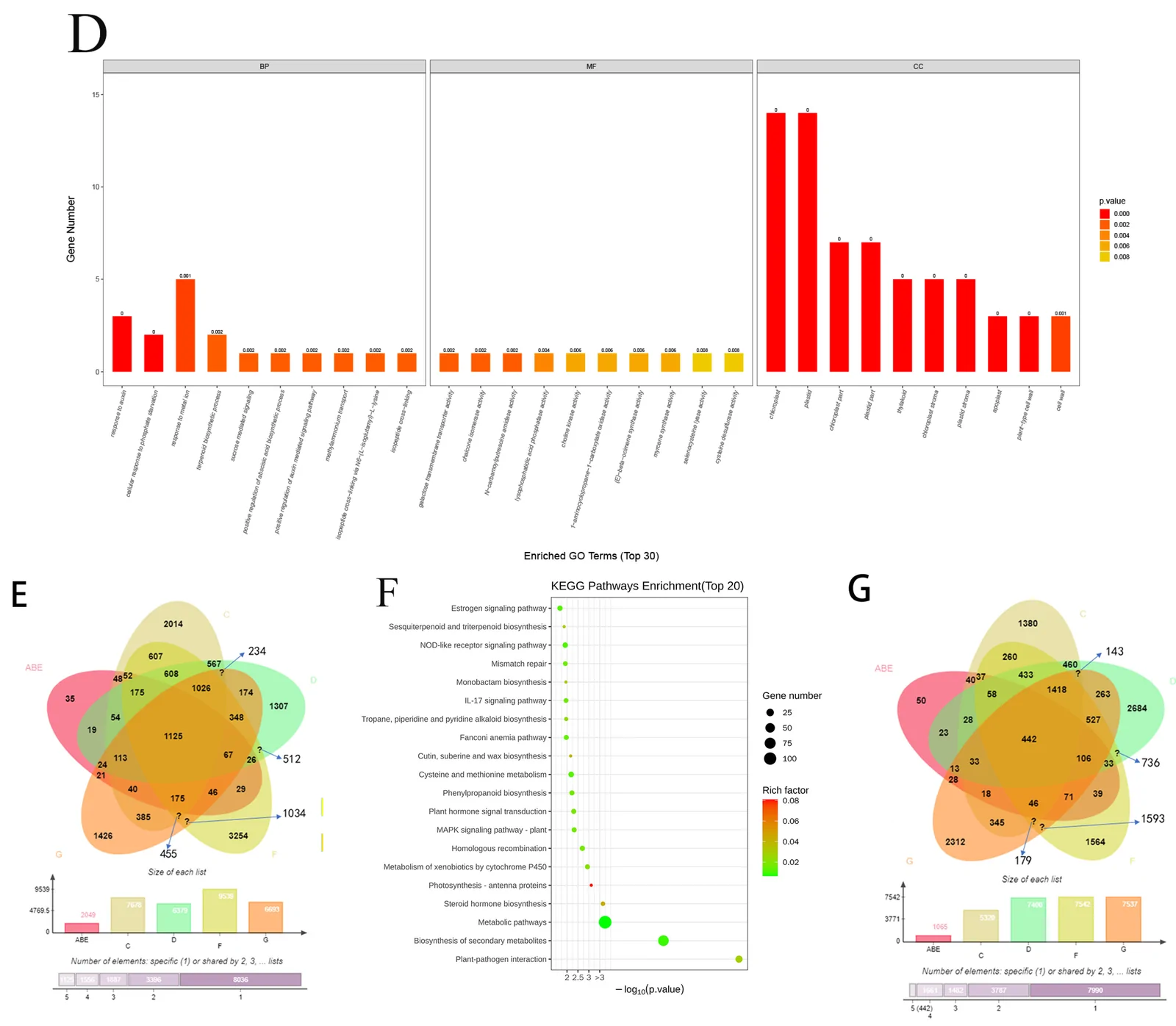
Identification and functional analysis of DEGs. A is a three-stage (**A**,**B**,**E**) DEG downregulation Venn diagram, B is a three-stage (**A**,**B**,**E**) DEG upregulation Venn diagram, (**C**) is a directed acyclic graph of the cellular components of the seven stages of DEG GO functional annotation, (**D**) is a GO-annotated Level 2 classification chart (Top 30), (**E**) is a seven-stage DEG upregulation Venn diagram, (**F**) shows the seven-stage DEG KEGG Pathways Enrichment (Top 20), and (**G**) is a seven-stage DEG downregulation Venn diagram.

Analysis of differential gene expression between SL and DL across the seven stages showed a total of 106,198 DEGs. The highest number of DEGs occurred at stage E. According to the differential significance analysis criteria, a lower adjusted pvalue and padj indicates more significant gene expression differences. Stages A and E had the highest number of DEGs, with pvalue and padj equal to zero (205 at stage A and 229 at stage E). The statistical analysis of the DEGs between SL and DL showed that there were 2049 upregulated (Figure 7B) and 1065 downregulated (Figure 7A) DEGs shared at stages A, B, and E. In the seven stages, 1125 genes were upregulated (Figure 7D) and 442 were downregulated (Figure 7G).

GO enrichment analysis of the DEGs shared by SL and DL at the seven stages revealed that biological processes (GO:0008150) were enriched in 38 DEGs, with cellular process (GO:0009987) having the highest DEG count (25). For molecular function (GO:0003674), 42 DEGs were enriched, with binding (GO:0003674) having the highest DEG count (27 DEGs). Cellular components (GO:0005575) were enriched in 55 DEGs, with cell part (GO:0044464) being the most frequent (49 DEGs). In the GO enrichment results, the most significant differences between *Isatis* varieties SL and DL occurred in cellular components. A directed acyclic graph (Figure 7C) of cellular component DEGs showed high enrichment in plastid (GO:0009536) and chloroplast (GO:0009507), with both containing 14 identical genes. The final node in the directed acyclic graph converged at chloroplast stroma (GO:0009570), with five DEGs enriched, suggesting that the primary differences between the leaf tissues of *Isatis* varieties SL and DL are focused on the chloroplast. In the NR database comparison and annotation results, the accession IDs of the 14 DEGs enriched in GO terms GO:0009536, GO:0009507, and GO:0009570 helped retrieve related protein and gene information from the NCBI database. The Hit1_eValue was calculated based on the Score, Query sequence length, and database size. Evalue essentially represents the false-positive rate, with a smaller E-value indicating a lower false-positive rate. NCBI search results showed that only 10 of the 14 DEGs had corresponding genes in the database. Among them, five genes were found in Arabidopsis thaliana (*TPS-CIN*, Gene ID: 822175; *AT5G36790*, Gene ID: 833646; *PDIL1-1*, Gene ID: 838779; *APS3*, Gene ID: 827118; *CCH*, Gene ID: 824790), one in Arabidopsis lyrata subsp (*LOC9317977*, Gene ID: 9317977), one in lyrata (*LOC9317977*, Gene ID: 9317977), two in Eutrema salsugineum (*LOC18994026*, Gene ID: 18994026; *LOC18022161*, Gene ID: 18022161), and two in Camelina sativa (*LOC104730901*, Gene ID: 104730901; *LOC104719239*, Gene ID: 104719239).

KEGG pathway enrichment analysis was conducted on the DEGs shared by SL and DL in the seven stages (Figure 7F). A total of 228 DEGs were annotated to 201 predicted KEGG metabolic pathways. Referring to previous research [25], two pathways related to indole metabolism were identified: tryptophan metabolism (ko00380) and phenylalanine, tyrosine, and tryptophan biosynthesis (ko00400). These pathways had four mapped DEGs (*TR21126_c0_g1*, *TR14351_c0_g1*, *TR476_c1_g3*, and *TR14696_c0_g1*) and two mapped DEGs (*TR2633_c1_g2* and *TR1952_c4_g1*), respectively. Additionally, within the ko00380 pathway, cytochrome P450 family 1 subfamily A1 and catalase were noted to be upregulated, while in the ko00400 pathway, 3-phosphoshikimate 1-carboxyvinyltransferase and tyrosine aminotransferase were also upregulated. The accession IDs for DEGs *TR21126_c0_g1*, *TR14351_c0_g1*, *TR476_c1_g3*, *TR14696_c0_g1*, *TR2633_c1_g2*, and *TR1952_c4_g1* were queried in the comparison and annotation results using the NR database. The corresponding protein and gene-related information were queried in the NCBI database according to the accession ID. The NCBI database query results showed that only one DEG (*LOC106390999*, Gene ID: 106390999) had related information and was found in Brassica napus.

The analysis of the top 20 biosynthesis and metabolic pathways showed that the pathway with the highest number of enriched DEGs was metabolic pathways (ko01100) and the most significantly different pathway was plant–pathogen interaction (ko04626). The pathway with the highest richness factor was photosynthesis-antenna proteins (ko00196), followed by biosynthesis of secondary metabolites (ko01110), cysteine and methionine metabolism (ko00270), and metabolism of xenobiotics by cytochrome P450 (ko00980). Upregulated enzymes in pathways ko04626, ko00196, ko00270, and ko00980 included CPK, CALM, MEKK1, SUGT1, HSP90B, RPS2, EDS1, CTSF, KCS, LHCA3, LHCB1, PHGDH, MDH, E2.6.1.42, LDH, DNMT1, MAT, AMD1, E1.14.17.4, TAT, CYP1A1, and GST.

#### 3.5.4. qRT-PCR Validation Analysis

**Figure 8:**
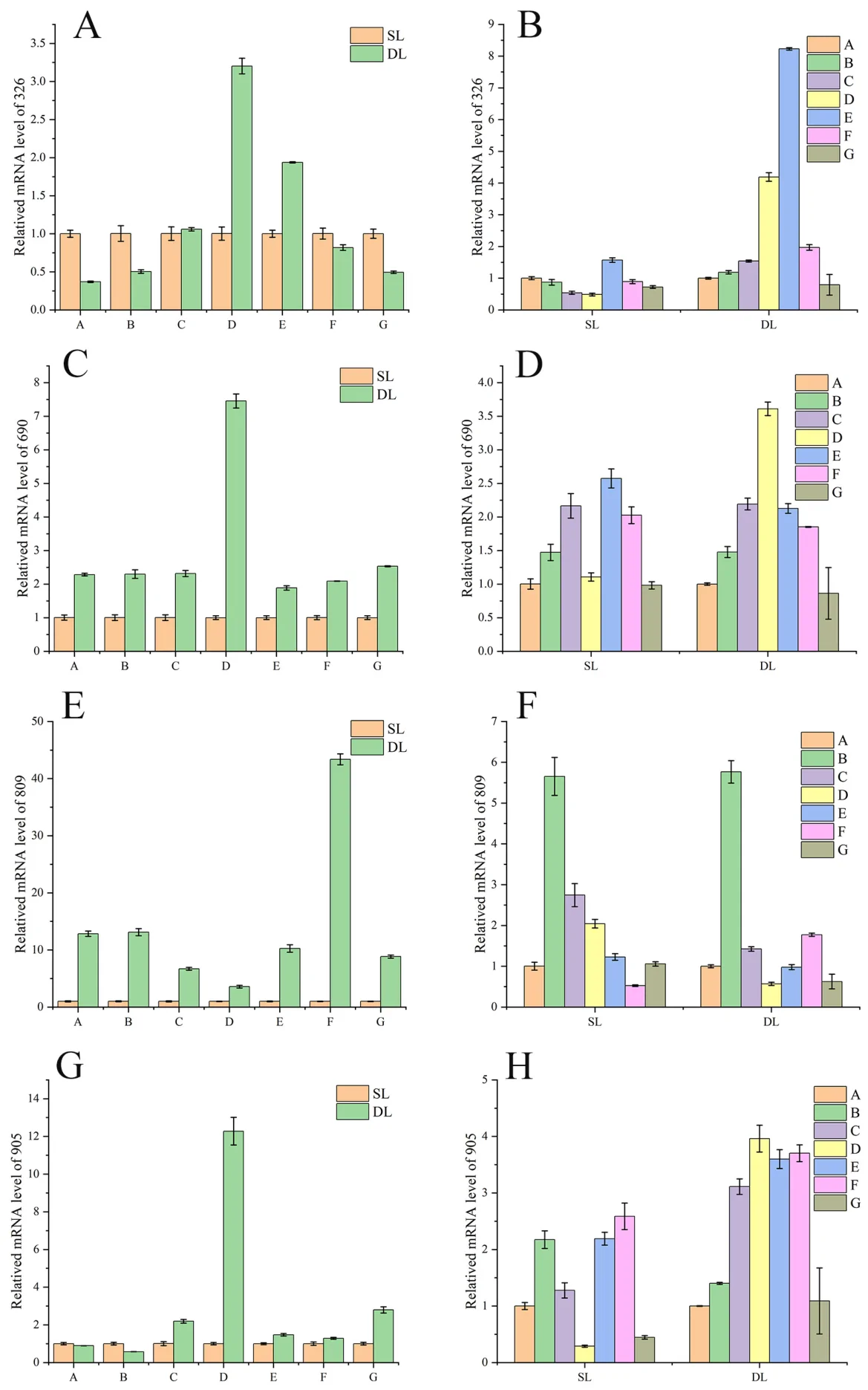
qRT-PCR validation analysis results. The 2^−ΔΔCT^ results of TR24326_c0_g1 at different developmental stages (**A**,**B**); the 2^−ΔΔCT^ results of TR37690_c0_g1 at different developmental stages (**C**,**D**); the 2^−ΔΔCT^ results of TR809_c0_g1 at different developmental stages (**E**,**F**); the 2^−ΔΔCT^ results of TR37905_c0_g1 at different developmental stages (**G**,**H**).

To verify the reliability of the transcriptome data, four candidate transcripts, namely *TR24326_c0_g1*, *TR37690_c0_g1*, *TR37905_c0_g1*, and *TR809_c0_g1*, were selected for quantitative real-time polymerase chain reaction (qRT-PCR) validation. The data from transcriptome sequencing and qRT-PCR for these candidate transcripts were transformed using 2^−ΔΔCT^ and the expression trends from both techniques were compared. The results showed that the expression trends were consistent between the two methods (Figure 8), proving the reliability of the RNA-seq results.

### 3.6. Metabolome and Transcriptome Correlation Analysis

**Figure 9:**
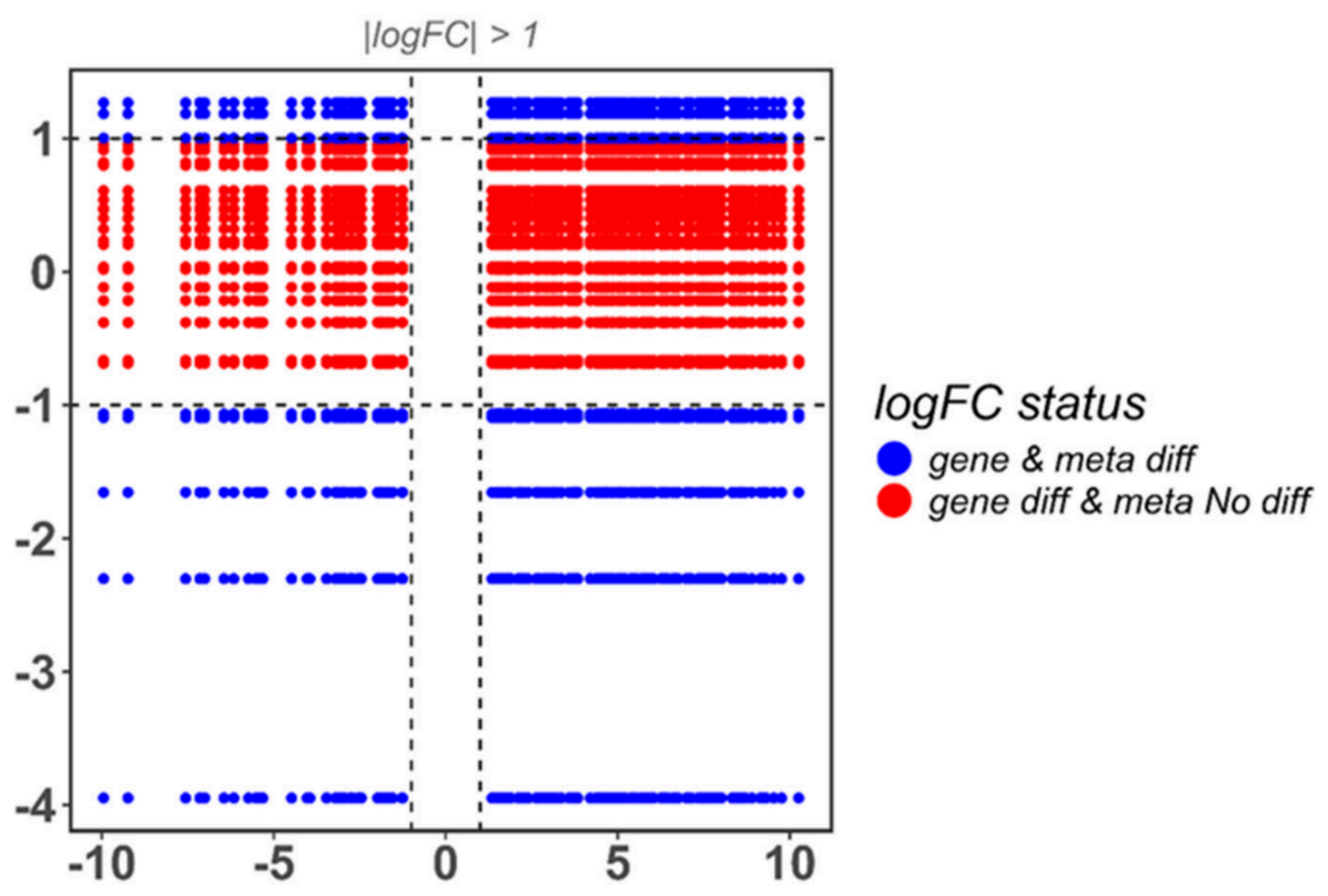
Nine-quadrant plot of DEGs and DEMs.

Based on the metabolomic analysis results, 26 differentially expressed metabolites (DEMs) were identified at stages A, B, and E, which were either upregulated or downregulated when comparing SL and DL. Based on the transcriptomic analysis results in the seven stages, 31 DEGs were identified in the GO top 30 and 139 in the KEGG top 20, totaling 170 DEGs. Using R, a correlation analysis of the 26 DEMs and 170 DEGs was conducted using a nine-quadrant plot (Figure 9). The DEMs and DEGs showed a positive correlation in Quadrant 7 and negative correlation in Quadrant 9. There were 17 DEMs correlated with DEGs across stages A, B, and E: isoorientin, peonidin-3-glucoside, isorhamnetin 3-galactoside, 8-[3,4-dihydroxy-5-(hydroxymethyl)oxolan-2-yl]-5,7-dihydroxy-2-(4-hydroxyphenyl)-6-[3,4,5-trihydroxy-6-(hydroxymethyl)oxan-2-yl]chromen-4-one, tiliroside, apigenin-6-C-glucoside-7-O-glucosid, violanone, 2,6-dihydroxy-2-(4-hydroxybenzyl)-3-oxo-2,3-dihydro-1-benzofuran-4-yl 6-O-(6-deoxy-alpha-L-mannopyranosyl)-beta-D-glucopyranoside, 3-[(2S,3R,4S,5S,6R)-4,5-dihydroxy-6-(hydroxymethyl)-3-[(2S,3R,4S,5R,6R)-3,4,5-trihydroxy-6-(hydroxymethyl)oxan-2-yl]oxyoxan-2-yl]oxy-5-hydroxy-2-(4-hydroxyphenyl)-7-methoxychromen-4-one, FA 16:0;2O, isoscoparin, tectoridin, indigo, sinapoy-lagmatine, camptothecin, dimethyl 2-[3-(4-hydroxy-3,5-dimethoxyphenyl)prop-2-enoyloxy]butanedioate, and xi-3-hydroxy-5-phenylpentanoic acid O-beta-D-glucopyranoside. Four DEGs correlated with DEMs at stages A, B, and E were identified: TR319_c0_g1, TR2281_c0_g1, TR510_c0_g1, and TR55919_c0_g2. TR510_c0_g1 and TR55919_c0_g2 were annotated to 250 functional terms in the GO database, with 37 of these terms related to cellular components. Shared GO annotations for these two genes included “cytoplasmic part” (GO:0044444), “cytoplasm” (GO:0005737), “organelle” (GO:0043226), “intracellular organelle” (GO:0043229), “membrane-bounded organelle” (GO:0043227), and others, totaling 11 terms. The KEGG pathway annotation for TR510_c0_g1 and TR55919_c0_g2 showed an association with three pathways, namely biosynthesis of secondary metabolites (ko01110), metabolic pathways (ko01100), and monoterpenoid biosynthesis (ko00902). By querying the NR database, the accession IDs for TR510_c0_g1 and TR55919_c0_g2 (NP_001326843.1 and 4PYG_A) were obtained. A further search in the NCBI database showed that only TR510_c0_g1 was linked to the gene TPS-CIN (Gene ID: 822175) and its associated proteins, terpene synthase-like sequence-1,8-cineole and protein-glutamine γ-glutamyltransferase.

## 4. Discussion

### 4.1. Study on Differences in Phenotypic Traits and Photosynthetic Characteristics of Different I. indigotica Varieties

Morphological characteristics represent the most direct and visible manifestation of genetic divergence in plants. Phenotypic variation reliably mirrors intrinsic genetic diversity and serves as a crucial evaluation index for germplasm resource screening and crop breeding, which has been extensively documented in previous studies [26]. In the present study, two cultivated *I. indigotica* varieties (SL and DL) were comparatively analyzed in terms of multiple agronomic and physiological traits. We quantified plant height, plant width, leaf length, leaf width, leaf number, and single-plant leaf fresh and dry weights, and further characterized leaf morphological structures and dynamic accumulation patterns of chlorophyll a, chlorophyll b, and carotenoids across different growth stages. Significant phenotypic differences in leaf architecture were identified between the two varieties. Specifically, SL produces obovate leaves with wrinkled and undulated blades, and the leaf surface is covered with a thick wax powder layer. In contrast, DL exhibits oblanceolate leaves with smooth and flat leaf surfaces without undulation and wax powder deposition. From the perspective of medicinal cultivation and industrial production, SL possesses superior leaf morphological traits and higher leaf yield, thereby providing better economic benefits. These favorable agronomic characteristics simplify field cultivation and post-harvest processing, enabling the stable acquisition of high-quality medicinal raw materials, which makes SL more suitable for large-scale popularization and application compared with DL.

Visible disparities in leaf color between SL and DL further reflected genotype-dependent variations in photosynthetic pigment pools. It is generally recognized that sun-adapted plants normally maintain a chlorophyll a/b ratio close to 3:1, while shade-tolerant species typically exhibit a ratio of approximately 2.3:1 [27]. Our pigment quantification revealed that the chlorophyll a/b ratios of SL and DL leaves reached 2.1–2.3 at stage A and gradually approached 3 in subsequent developmental stages. This developmental trend indicated a progressive increase in the relative abundance of chlorophyll a versus chlorophyll b from stage A to stage G, implying that *I. indigotica* is predominantly adapted to absorb long-wavelength red light. Nevertheless, it should be noted that chlorophyll a/b ratio alone cannot provide definitive evidence for thermophilic adaptation; targeted controlled temperature experiments are required to further verify thermal responsiveness. Collectively, pigment characteristics support the classification of *I. indigotica* as a sun-loving species.

Carotenoids function as multifunctional photoprotective pigments: they transfer captured light energy to chlorophyll, quench excited-state chlorophyll molecules, and dissipate surplus excitation energy via the xanthophyll cycle, thereby mitigating photooxidative damage to the photosynthetic apparatus under elevated irradiance. In the current study, leaf carotenoid concentrations increased markedly in SL at stages B and G, whereas significant carotenoid accumulation was detected in DL at stages D and G. Differential timing of carotenoid upregulation between the two varieties may reflect genotypic divergence in photoprotective responses. Given that this experiment was conducted under natural field light regimes without controlled high-light treatments, we cautiously infer that SL may be more susceptible to light-induced stress, which warrants validation via dedicated light-manipulating trials.

### 4.2. Analysis of Differences in the AsA-GSH Antioxidant System, Key Photosynthetic Enzymes, and Chloroplast-Related Metabolism

The ascorbic acid–glutathione (AsA–GSH) cycle, also termed the Foyer–Halliwell–Asada pathway, constitutes the core enzymatic and non-enzymatic antioxidant system responsible for hydrogen peroxide detoxification in plant chloroplasts and cytosol, and it maintains intracellular redox homeostasis during photosynthetic metabolism and abiotic stress responses [28,29]. This cyclic pathway precisely coordinates multiple antioxidant substances and functional enzymes, efficiently eliminating excess reactive oxygen species (ROS) generated by electron leakage during light reactions and preventing oxidative damage to thylakoid membranes and photosynthetic apparatus [30]. The core components of the AsA–GSH system include two key non-enzymatic antioxidants (ascorbic acid, AsA; reduced glutathione, GSH) and four rate-limiting enzymes: ascorbate peroxidase (APX), monodehydroascorbate reductase (MDHAR), dehydroascorbate reductase (DHAR), and glutathione reductase (GR) [31]. AsA directly quenches H_2_O_2_ and singlet oxygen to alleviate photooxidative stress [32], while GSH acts as an essential reducing substrate for DHAR and glutathione peroxidase (GSH-Px), indirectly participating in ROS scavenging and redox balance maintenance [33].

APX catalyzes the oxidation of AsA by H_2_O_2_ to form monodehydroascorbate, whereas SOD serves as the first line of antioxidant defense, dismutating superoxide anion radicals into H_2_O_2_ and O_2_ [34,35]. GR mediates the NADPH-dependent regeneration of GSH from oxidized glutathione (GSSG), and GSH-Px further consumes GSH to eliminate toxic ROS, thereby jointly stabilizing membrane structure and sustaining normal cellular metabolism [36]. In the present field-based study, significantly higher SOD activity was detected in SL leaves at stages B, C, D, F, and G, while GR activity was consistently and markedly elevated in SL across all seven developmental stages. Combined with stage-specific increases in APX and GSH-Px activities in SL, these findings demonstrate that SL possesses a more robust antioxidant enzyme system. Notably, higher antioxidant enzyme activity does not exclusively indicate greater stress sensitivity; it can also reflect enhanced intrinsic ROS detoxification and adaptive capacity. The superior AsA–GSH antioxidant capacity in SL may represent a constitutive adaptive strategy to cope with fluctuating natural light conditions rather than merely a stress-sensitive phenotype.

Photosynthetic carbon assimilation efficiency is jointly determined by antioxidant status and key carbon-fixation enzymes. Ribulose-1,5-bisphosphate carboxylase/oxygenase (Rubisco) and fructose-bisphosphate aldolase (FBA) are rate-limiting enzymes in the Calvin cycle, whose activities directly determine carbon-fixation efficiency and photosynthetic productivity. In this study, SL exhibited significantly higher Rubisco and FBA activities than DL, consistent with the higher SBPase and TK activities detected in SL. Combined with the superior leaf morphological traits and higher photosynthetic pigment accumulation in SL, we speculate that the differential chloroplast physiological status between the two varieties modulates carbon-fixation enzyme activity. These physiological discrepancies further validate the transcriptomic results that genotype-specific transcriptional variations between SL and DL are predominantly enriched in chloroplast metabolism pathways.

### 4.3. Comparison of Indirubin Accumulation Characteristics in Leaves of Different I. indigotica Varieties and Analysis of Medicinal Potential

Quantitative comparison of characteristic active ingredients is the core basis for evaluating germplasm quality and screening superior medicinal plant varieties, as secondary-metabolite accumulation directly determines the medicinal value of herbal materials [37]. Previous cross-species comparisons of indigo medicinal plants have confirmed significant interspecific differences in indigo and indirubin contents among *I. indigotica*, Polygonum tinctorium, and Baphicacanthus cusia, with *I. indigotica* accumulating the highest levels of indole alkaloids, indicating its unique advantages in medicinal material production [38]. Interspecific and intraspecific metabolite variations are primarily governed by genotype, followed by developmental stage and environmental fluctuation.

In the present intraspecific variety comparison, dynamic detection of medicinal ingredients showed that SL maintained a significantly higher indirubin content than DL throughout all seven growth stages. The most prominent differences occurred in the early vegetative stages: the indirubin content of SL was 2.57-fold and 2.39-fold higher than that of DL at stages A and B, respectively. Although the amplitude of varietal difference gradually narrowed in the middle and late growth stages (C–G), SL consistently retained higher indirubin accumulation than DL. This stage-dependent dynamic variation indicates that the genotypic advantage of SL in indole alkaloid biosynthesis is most prominent in young leaves, which provides a theoretical basis for optimizing the harvesting period of high-quality *I. indigotica* medicinal materials. Considering the higher leaf yield and superior leaf architecture of SL, this variety exhibits comprehensive advantages in the production of indirubin-enriched medicinal raw materials. Nevertheless, this study only determined the endogenous accumulation of active ingredients; further pharmacological experiments, including antioxidant, anti-inflammatory, and antiviral activity assays, are required to verify the practical medicinal efficacy of SL-derived herbal materials.

### 4.4. Analysis of Metabolomic Differences Among Different I. indigotica Varieties and the Mechanism of Anthocyanin-Mediated Adaptation to High Light

*I. indigotica* is a traditional Chinese medicinal herb with detoxification, anti-inflammatory, and antiviral pharmacological functions, which is widely used in the clinical prevention and treatment of influenza, mumps, and viral hepatitis [39,40,41]. Indole alkaloids are recognized as the core bioactive components of *I. indigotica*, whose biosynthesis originates from the indole-3-glycerol phosphate metabolic pathway, with indoxyl serving as the key precursor for indigo and indirubin synthesis [42,43,44]. In addition to indole alkaloids, flavonoids and anthocyanins are crucial secondary metabolites affecting plant stress adaptation and medicinal quality.

In this study, a total of 1217 metabolites were identified via widely targeted metabolomics covering positive and negative ionization modes. Since partial metabolite overlap exists between dual-mode identification, the 1217 metabolites represent the total detected signals rather than unique compounds; subsequent differential analysis was performed based on unique annotated metabolites to avoid statistical bias. A total of 115 commonly upregulated metabolites and 169 total differential metabolites (DEMs) were identified in SL compared with DL, among which only 39 consistent DEMs were shared across the three key developmental stages (A, B, and E). Classification analysis revealed that flavonoids were the most enriched differential metabolites, accounting for 11 of the 26 stably varied metabolites, including apigenin-6-C-glucoside-7-O-glucoside, isoorientin, cosmosiin, and peonidin-3-glucoside. These flavonoid compounds have been widely reported to possess antioxidant and photoprotective functions, and can be used as potential metabolic markers for germplasm distinction of *I. indigotica* varieties.

Notably, peonidin-3-glucoside, a core differential anthocyanin compound annotated to the anthocyanin biosynthesis pathway, exhibited significantly higher accumulation in SL at stages A, B, and E. Anthocyanins function as vital photoprotective substances in plants by modulating light absorption spectra, filtering excess high-energy light, and scavenging ROS, thereby alleviating high-light-induced photoinhibition and protecting the photosynthetic system [45,46]. In this study, SL displayed significantly higher GSH-Px activity and lower GSSG content than DL at key growth stages, forming a more efficient GSH redox cycle. Although no significant statistical correlation analysis was performed between peonidin-3-glucoside abundance and GSH-Px/GSSG levels in the current dataset, the consistent variation trend implies a potential synergistic relationship between anthocyanin accumulation and the antioxidant enzyme system in SL. Combined with the stage-specific carotenoid accumulation characteristics, we cautiously hypothesize that the enriched peonidin-3-glucoside in SL may assist in mitigating high-light stress, thereby maintaining a stable photosynthetic performance under natural fluctuating light conditions. Further quantitative detection of total anthocyanin content, Fv/Fm, ΦPSII, NPQ, and ROS/MDA levels is required to solidify this regulatory mechanism.

### 4.5. Transcriptome Analysis of Key Genes and Chloroplast-Related Pathways Contributing to Leaf Differences Between SL and DL I. indigotica Varieties

Transcriptomic profiling across the seven developmental stages revealed widespread genotypic transcriptional divergence between SL and DL. A total of 782 persistently upregulated and 443 persistently downregulated genes were stably differentially expressed in SL relative to DL. GO functional enrichment demonstrated that the differential genes were predominantly enriched in chloroplast-related cellular component terms, indicating that chloroplast metabolism variation is the core genetic basis for leaf phenotypic divergence between the two varieties. Ten high-confidence chloroplast-related DEGs with complete NCBI functional annotation were screened for subsequent mechanism analysis, excluding four genes with incomplete database matching information.

*LOC18994026* (*LCD1*) was stably and significantly upregulated in SL throughout all growth stages. Previous studies have verified that *LCD1* negatively regulates leaf senescence by inhibiting chlorophyll degradation in model plants [47]. In the present study, the high expression of *LCD1* in SL was accompanied by a higher chlorophyll content, suggesting a potential inhibitory effect on chlorophyll degradation. However, this study only confirms a correlational relationship; the expression levels of classic chlorophyll degradation genes (*NYC1*, *PAO*, *PPH*, and *SGR1*) were not quantified. Therefore, we cannot definitively conclude that *LCD1* directly suppresses the chlorophyll degradation pathway in *I. indigotica*. The observed higher chlorophyll retention in SL may be comprehensively regulated by multiple senescence-related genes.

*LOC104719239* (*AK5*), encoding adenylate kinase 5, was consistently upregulated in SL. Adenylate kinase participates in intracellular adenine nucleotide balance regulation, mediating the reversible conversion of ATP, ADP, and AMP, and providing energy substrates for photosynthetic carbon fixation [48]. Combined with the higher photosynthetic pigment content and elevated Rubisco/FBA activities in SL, we speculate that high *AK5* expression may contribute to improved photosynthetic efficiency. Nevertheless, the current evidence remains correlative: the increased *AK5* expression may be a physiological consequence of superior photosynthetic performance in SL, rather than a direct causal regulatory factor. Subsequent detection of ATP/ADP/AMP energy status and genetic verification experiments are required to clarify its specific regulatory function.

KEGG enrichment of the top 20 metabolic pathways identified 22 key differentially expressed enzymes in SL, which were classified into stress response, immune regulation, photosynthetic carbon fixation, and cell cycle regulation categories. These functional annotations further confirm that varietal differences in leaf growth and physiological adaptation are mainly derived from differential chloroplast metabolic and stress signaling pathways.

### 4.6. Integrated Transcriptome and Metabolome Analysis of Differential Metabolic Pathways and Putative Differences in Cold Tolerance Between I. indigotica Varieties

Nine-quadrant integrated transcriptome–metabolome correlation analysis was performed on 26 core differential metabolites and 170 stage-stable DEGs. A total of 17 metabolites exhibited synchronous variation with DEGs across stages A, B, and E, while only four DEGs showed a paired correlation with these metabolites. Most correlated metabolites belonged to the phenylpropanoid and flavonoid superfamily, consistent with the metabolomic finding that flavonoid metabolism is the most divergent pathway between SL and DL.

In this study, a relatively strict correlation threshold of Pearson’s r > 0.9 was adopted to screen highly correlated gene–metabolite pairs, aiming to filter biologically meaningful associations and reduce false-positive correlations caused by high-throughput omics data noise. However, a high positive correlation only reflects synchronous variation trends and cannot prove mutual positive regulation or causal interaction. Without transgenic verification or in vitro functional experiments, the correlation results can only be used for preliminary mechanism speculation rather than definitive regulatory conclusion.

Among the four core correlated DEGs, *LOC18029281* encodes phospho-2-dehydro-3-deoxyheptonate aldolase 2, a key rate-limiting enzyme in the tryptophan biosynthesis pathway. Previous studies have shown that enhanced tryptophan metabolism contributes to plant cold tolerance by regulating osmotic balance and stress signaling [49]. In this study, DL exhibited higher *LOC18029281* expression and higher endogenous tryptophan accumulation than SL. During stage G, the field ambient temperature gradually decreased from 10 °C to 0 °C under natural cultivation conditions, with consistent humidity, soil fertility, and management measures. Phenotypic observation showed that SL leaves exhibited earlier wilting and senescence symptoms under low-temperature conditions, implying that DL may have better cold acclimation ability. It should be noted that this cold tolerance judgment is based on natural low-temperature phenotypic observation and metabolic/gene expression correlation; quantitative low-temperature stress experiments and tryptophan downstream metabolite determination are still needed to verify the cold resistance regulatory mechanism of *LOC18029281*-mediated tryptophan metabolism.

## 5. Conclusions

The two *I. indigotica* varieties SL and DL exhibited significant differences in morphological traits, photosynthetic physiology, antioxidant metabolism, medicinal ingredient accumulation, and multi-omics profiles. From the perspective of medicinal cultivation and industrial production, SL has prominent application advantages, with a superior leaf morphological structure, thicker leaf wax powder layer, higher leaf yield, and consistently higher indirubin content throughout growth stages, making it an excellent germplasm resource for high-quality medicinal material production.

Physiologically, SL possesses a more robust AsA–GSH antioxidant system with higher SOD, GR, APX, and GSH-Px enzyme activities, which endows it with a stronger intrinsic ROS scavenging and oxidative stress adaptation capacity rather than merely high light stress sensitivity. The genotype-specific accumulation of peonidin-3-glucoside in SL may cooperate with the antioxidant system to alleviate high-light-induced photoinhibition, maintaining a stable photosynthetic performance under natural field conditions. Transcriptomic differences between the two varieties were mainly enriched in chloroplast metabolism pathways. The high expression of *LCD1* (*LOC18994026*) in SL is correlated with reduced chlorophyll degradation and improved photosynthetic retention capacity, while elevated *AK5* (*LOC104719239*) expression may facilitate energy metabolism and carbon-fixation efficiency, collectively promoting the superior agronomic performance of SL.

This study systematically clarifies the phenotypic, physiological, and molecular basis of varietal differences in *I. indigotica*, provides theoretical support for the selective cultivation of superior medicinal varieties, and lays a foundation for further exploration of stress adaptation mechanisms and medicinal quality improvement of *I. indigotica*.

## Figures and Tables

**Table 1:** Sample information for seven developmental stages of *I. indigotica* cultivars SL and DL.

Serial No.	Sampling Date	Time No.	S Sample No.	D Sample No.	Days After Emergence	Morphological Criteria
1	8 August 2024	A	SLA	DLA	71	Rosette–leaf vigorous-expansion stage
2	23 August 2024	B	SLB	DLB	86	Rapid vegetative-growth stage
3	8 September 2024	C	SLC	DLC	102	Late vegetative-growth stage
4	23 September 2024	D	SLD	DLD	117	Early mature rosette stage
5	8 October 2024	E	SLE	DLE	132	Peak secondary-metabolite accumulation stage
6	23 October 2024	F	SLF	DLF	147	Late mature stage
7	8 November 2024	G	SLG	DLG	163	Senescence-onset stage

Note: Sampling time was based on the Chinese 24-Solar-Terms calendar. Developmental staging was determined primarily based on morphological traits rather than calendar date, to align phenological status between SL and DL.

**Table 2:** PCR primer sequences.

Primer Name	Nucleotide Sequence (5′-3′)		Product Length (bp)
TR24326_c0_g1	sense	AAGTAGCGGTGGGTGGTATGT	81
	antisense	TTGGGGAAAACAGTGGAAGAC	
TR37690_c0_g1	sense	ATCGCTCAGCTCACCCAAAA	105
	antisense	CGAAACGAAGACCCGAGGA	
TR37905_c0_g1	sense	CAAACACCAACCTCTTTCCCTT	251
	antisense	GTTTCGTTCTTCCTCCTTCTCC	
TR809_c0_g1	sense	GACCGCTTTCTGGTTCTGGA	106
	antisense	AGAATCTCCTCCGCTACACTCC	
Actin	sense	GGAGAAGAACTACGAGCTA	145
	antisense	GCTGGAAGAGTATTTCAGG	

## Data Availability

The raw experimental data supporting the findings of this study are openly available on Zenodo at https://doi.org/10.5281/zenodo.21818697 (accessed on 15 August 2026).

## Abbreviations

The following abbreviations are used in this manuscript:

SL	Songming No. 1
DL	Dinglan No. 1
APX	ascorbate peroxidase
GR	glutathione reductase
GSH-Px	glutathione peroxidase
SOD	superoxide dismutase
SBPase	sedoheptulose-1,7-bisphosphatase
FBA	fructose-1,6-bisphosphate aldolase
TK	transketolase
GSH	glutathione
GSSG	oxidized glutathione
DEGs	differentially expressed genes
DEMs	differentially expressed metabolites

## References

[1] Santiago D., Cunha J., Cabral I. (2023). Chromatic and medicinal properties of six natural textile dyes: A review of eucalyptus, weld, madder, annatto, indigo and woad. Heliyon.

[2] Garcia-Macias P., John P. (2004). Formation of natural indigo derived from woad (*Isatis tinctoria* L.) in relation to product purity. J. Agric. Food Chem..

[3] Alper C., İlknur A., Tunahan D., Nilgün G.B. (2008). Alpha-tryptophan synthase of *Isatis tinctoria*: Gene cloning and expression. Plant Physiol. Biochem..

[4] Spataro G., Taviani P., Negri V. (2007). Genetic variation and population structure in a Eurasian collection of *Isatis tinctoria* L.. Genet. Resour. Crop Evol..

[5] Cettina C., Antonella V., Vincenza R., Marisa Z., Alessandra T., Salvatore R. (2006). The leaf volatile constituents of *Isatis tinctoria* by solid-phase microextraction and gas chromatography/mass spectrometry. Planta Med..

[6] Naganuma M. (2019). Treatment with indigo naturalis for inflammatory bowel disease and other immune diseases. Immunol. Med..

[7] Mohan S.G., Alex V.V., Amrutha N.A., Bava S.V., Sundaram S., Retnakumari A.P., Sadasivan C., Anto R.J. (2020). Pre-clinical evidences for the efficacy of tryptanthrin as a potent suppressor of skin cancer. Cell Prolif..

[8] Yang L., Li X.Y., Huang W., Rao X.S., Lai Y. (2022). Pharmacological properties of indirubin and its derivatives. Biomed. Pharmacother..

[9] Wang P., Liu D., Yang F.H., Ge H., Zhao X., Chen H.G., Du T. (2021). Identification of key gene networks controlling vernalization development characteristics of *Isatis indigotica* by full-length transcriptomes and gene expression profiles. Physiol. Mol. Biol. Plants.

[10] An Y.W., Yuan B., Wang J.C., Wang C., Liu T.T., Song S., Liu H.Q. (2021). Clinical characteristics and impacts of traditional Chinese medicine treatment on the convalescents of COVID-19. Int. J. Med. Sci..

[11] An X.D., Zhang Y.H., Duan L.Y., Jin D., Zhao S.H., Zhou R.R., Duan Y.Y., Lian F.M., Tong X.L. (2021). The direct evidence and mechanism of traditional Chinese medicine treatment of COVID-19. Biomed. Pharmacother..

[12] Yang F.H., Zhao X., Wang P., Liu D., Chen H.G., Wang H.Z., Du T. (2022). Evaluation of germplasm resources of *Isatis indigotica*. Chin. Tradit. Herb. Drugs.

[13] Beloa A.P.M., Morgado C.M.A., Souza E.R.B.D., Ogata T., Pereira C.C.D.O., Junior L.C.C. (2018). Comparative and organic analysis and characterization of varieties of tangerines. Sci. Hortic..

[14] Kőrösi L., Molnár S., Teszlák P., Dörnyei Á., Maul E., Töpfer R., Marosvölgyi T., Szabó É., Röckel F. (2022). Comparative study on grape berry anthocyanins of various teinturier varieties. Foods.

[15] Xu C.P., Gui Z.H., Huang Y.X., Yang H.Z., Luo J., Zeng X.Q. (2023). Integrated transcriptomics and metabolomics analyses provide insights into Qingke in response to cold stress. J. Agric. Food Chem..

[16] Li J., Li Y.Q., Li M., Lin L.H., Qi J.M., Xu J.T., Zhang L.W., Fang P.P., Tao A.F. (2022). Novel insights into anthocyanin synthesis in the calyx of roselle using integrated transcriptomic and metabolomic analyses. Int. J. Mol. Sci..

[17] Cessur A., Albayrak İ., Demirci T., Baydar N.G. (2023). Silver and salicylic acid-chitosan nanoparticles alter indole alkaloid production and gene expression in root and shoot cultures of *Isatis tinctoria* and *Isatis ermenekensis*. Plant Physiol. Biochem..

[18] Qu R.J., Cao Y.W., Tang X.Q., Sun L.Q., Wei L., Wang K.C. (2021). Identification and expression analysis of the WRKY gene family in *Isatis indigotica*. Gene.

[19] Zhao K., Pu Y.Y., Shi H.Z., Guo Q.S., Su Y., Yang F., Liu C., Du Y. (2023). The potential mechanism of response to light intensity in energy metabolism mediated by miRNA in *Isatis indigotica*. Gene.

[20] Qiu N.W., Wang X.S., Zhou F. (2018). A new method for fast extraction and determination of chlorophylls in natural water. Z. Naturforschung C J. Biosci..

[21] Chazaux M., Schiphorst C., Lazzari G., Caffarri S. (2021). Precise estimation of chlorophyll a, b and carotenoid content by deconvolution of the absorption spectrum and new simultaneous equations for Chl determination. Plant J..

[22] Zhao G.H., Li T., Qu X.Y., Zhang N.N., Lu M., Wang J. (2018). Optimization of ultrasound-assisted extraction of indigo and indirubin from *Isatis indigotica* Fort. and their antioxidant capacities. Food Sci. Biotechnol..

[23] Chen Y.L., Zhu X., Yu Y.J., Cai Y., Li Y., Duan G.L. (2012). Comparison of infrared-assisted extraction and other techniques for analysis of indigo and indirubin in leaves of *Isatis indigotica* Fort. J. Liq. Chromatogr. Relat. Technol..

[24] Blaženović I., Kind T., Ji J., Fiehn O. (2018). Software tools and approaches for compound identification of LC-MS/MS data in metabolomics. Metabolites.

[25] Salim V., Jarecki S.A., Vick M., Miller R. (2023). Advances in metabolic engineering of plant monoterpene indole alkaloids. Biology.

[26] Torday J.S., Miller W.B. (2016). Phenotype as agent for epigenetic inheritance. Biology.

[27] Wang Z. (2010). Plant Physiology.

[28] Singh R., Parihar P., Prasad S.M. (2020). Sulphur and calcium attenuate arsenic toxicity in Brassicaceae by adjusting ascorbate-glutathione cycle and sulphur metabolism. Plant Growth Regul..

[29] Foyer C.H., Kunert K. (2024). The ascorbate–glutathione cycle coming of age. J. Exp. Bot..

[30] Shan C.J., Wang B.S., Sun H.L., Gao S., Li H. (2020). H_2_S induces NO in the regulation of AsA-GSH cycle in wheat seedlings by water stress. Protoplasma.

[31] Wu Y., Hu L.L., Liao W.B., Mohammed M.D., Lu J., Xie J.M., Feng Z., Alejandro C.U., Yu J.H. (2019). Foliar application of 5-aminolevulinic acid (ALA) alleviates NaCl stress in cucumber (*Cucumis sativus* L.) seedlings through the enhancement of ascorbate-glutathione cycle. Sci. Hortic..

[32] Gill S.S., Tuteja N. (2010). Reactive oxygen species and antioxidant machinery in abiotic stress tolerance in crop plants. Plant Physiol. Biochem..

[33] Gabriella S., Tibor K., Gábor G., Gábor K. (2009). Glutathione as an antioxidant and regulatory molecule in plants under abiotic stress conditions. J. Plant Growth Regul..

[34] Wei H., Movahedi A.L., Chen J.X., Wang Y.Q., Liu G.Y., Yu C.M., Chen Y.H., Zhong F., Lian B.L., Zhang J. (2023). PtAPX9-PtLTPG14 modulates the AsA-GSH cycle for lipid mechanisms in poplar. Ind. Crops Prod..

[35] Tiwari M.K., Hägglund P.M., Møller I.M., Davies M.J., Bjerrum M.J. (2019). Copper ion/H_2_O_2_ oxidation of Cu/Zn-superoxide dismutase: Implications for enzymatic activity and antioxidant action. Redox Biol..

[36] Sikanyika M., Aragão D., McDevitt C.A., Maher M.J. (2019). The structure and activity of the glutathione reductase from Streptococcus pneumoniae. Acta Crystallogr. Sect. F Struct. Biol. Commun..

[37] Hassan E.M., El Gendy A.E.G., Abd-ElGawad A.M., Elshamy A.I., Farag M.A., Alamery S.F., Omer E.A. (2020). Comparative chemical profiles of the essential oils from different varieties of *Psidium guajava* L.. Molecules.

[38] Zhang Y.J., Zhu Y.Q., Chen J., Xia C., Deng J.L., Li H.J., Li Y.L., Li J., Liu P. (2018). Identification of three species commonly known as “daqingye” by internal leaf anatomy and high-performance liquid chromatography analyses. Acta Soc. Bot. Pol..

[39] Wu B.B., Wang L.Y., Jiang L.L., Dong L.L., Xu F.L., Lu Y.L., Jin J.H., Wang Z.Y., Liang G., Shan X.O. (2017). n-butanol extract from Folium isatidis inhibits the lipopolysaccharide-induced downregulation of CXCR1 and CXCR2 on human neutrophils. Mol. Med. Rep..

[40] Deng Y.P., Liu Y.Y., Liu Z., Li J., Zhao L.M., Xiao H., Ding X.H., Yang Z.Q. (2013). Antiviral activity of Folium isatidis derived extracts in vitro and in vivo. Am. J. Chin. Med..

[41] Liu Z., Yang Z.Q., Xiao H. (2010). Antiviral activity of the effective monomers from Folium isatidis against influenza virus in vivo. Virol. Sin..

[42] King R.R., Calhoun L.A. (2009). The thaxtomin phytotoxins: Sources, synthesis, biosynthesis, biotransformation and biological activity. Phytochemistry.

[43] Min G.Y., Kim T.I., Kim J.H., Cho W.K., Yang J.H., Ma J.Y. (2023). Anti-atopic effect of *Isatidis Folium* water extract in TNF-α/IFN-γ-induced HaCaT cells and DNCB-induced atopic dermatitis mouse model. Molecules.

[44] Maugard T., Enaud E., Choisy P., Legoy M.D. (2001). Identification of an indigo precursor from leaves of *Isatis tinctoria* (Woad). Phytochemistry.

[45] Mandell J.D., Diviti S., Xu M., Townsend J.P. (2024). Rare drivers at low prevalence with high cancer effects in T-cell and B-cell pediatric acute lymphoblastic leukemia. Int. J. Mol. Sci..

[46] Hu K.D., Peng X.J., Yao G.F., Zhou Z.L., Yang F., Li W.J., Zhao Y.Q., Li Y.Q., Han Z., Chen X.Y. (2021). Roles of a cysteine desulfhydrase LCD1 in regulating leaf senescence in tomato. Int. J. Mol. Sci..

[47] Wei W.K., Guo L.A., Tang X., Wang Y., Pan Y. (2026). CcFNSII-3-mediated metabolic partitioning and environmental regulation correlate with leaf-specific flavonoid accumulation for antioxidant potential in Coptis chinensis. Ind. Crops Prod..

[48] Giorgia B., Alessandra R., Salvatore E., Accursio V., Antonio L., Roberta N., Antonello C., Samuela P., Antonella V., Antonio M. (2024). Combined salt and low nitrate stress conditions lead to morphophysiological changes and tissue-specific transcriptome reprogramming in tomato. Plant Physiol. Biochem..

[49] Kwon H.H., Ahn C.H., Lee H.J., Sim D.Y., Park J.E., Park S.Y., Kim B., Shim B.S., Kim S.H. (2023). The apoptotic and anti-warburg effects of brassinin in PC-3 cells via reactive oxygen species production and the inhibition of the c-Myc, SIRT1, and β-catenin signaling axis. Int. J. Mol. Sci..

